# EpiReSIM: A Resampling Method of Epistatic Model without Marginal Effects Using Under-Determined System of Equations

**DOI:** 10.3390/genes13122286

**Published:** 2022-12-04

**Authors:** Junliang Shang, Xinrui Cai, Tongdui Zhang, Yan Sun, Yuanyuan Zhang, Jinxing Liu, Boxin Guan

**Affiliations:** 1 School of Computer Science, Qufu Normal University, Rizhao 276826, China; 2 Science and Technology Innovation Service Institution of Rizhao, Rizhao 276827, China; 3 School of Information and Control Engineering, Qingdao University of Technology, Qingdao 266520, China

**Keywords:** simulation, GWAS, epistasis model, resampling, penetrance table, prevalence, heritability

## Abstract

Simulation experiments are essential to evaluate epistasis detection methods, which is the main way to prove their effectiveness and move toward practical applications. However, due to the lack of effective simulators, especially for simulating models without marginal effects (eNME models), epistasis detection methods can hardly verify their effectiveness through simulation experiments. In this study, we propose a resampling simulation method (EpiReSIM) for generating the eNME model. First, EpiReSIM provides two strategies for solving eNME models. One is to calculate eNME models using prevalence constraints, and another is by joint constraints of prevalence and heritability. We transform the computation of the model into the problem of solving the under-determined system of equations. Introducing the complete orthogonal decomposition method and Newton’s method, EpiReSIM calculates the solution of the underdetermined system of equations to obtain the eNME model, especially the solution of the high-order model, which is the highlight of EpiReSIM. Second, based on the computed eNME model, EpiReSIM generates simulation data by a resampling method. Experimental results show that EpiReSIM has advantages in preserving the biological properties of minor allele frequencies and calculating high-order models, and it is a convenient and effective alternative method for current simulation software.

## 1. Introduction

With the completion of the human genome project and the rapid development of high-throughput sequencing technologies, tremendous amounts of genetic data, such as single nucleotide polymorphism (SNP) data, have been generated. There is a wealth of information in these data, and how to mine information in a large amount of data has become the key to many studies. Genome-wide association studies (GWAS) have emerged as a powerful way for discovering genetic variants associated with complex diseases, and GWAS often uses SNPs as genetic markers for case-control association studies.

The interaction between different genes when expressing a specific phenotype is called epistasis. The importance of epistasis in phenotypic-genotype associations has been established. Epistasis can be defined in different ways. Here we focus on and significantly expand upon statistical epistasis which is the deviation from additivity in mapping multi-locus genotypes to phenotypic variation [1]. To analyze epistatic interactions between SNPs, several epistasis detection methods have been proposed [2,3,4]. Although using a real biological SNP dataset is necessary, the real process behind it is usually unknown. Therefore, it is very complicated to evaluate the accuracy of these epistatic detection methods by using real biological SNP datasets [5]. The use of simulated data provides a new way to test the accuracy of the SNP epistasis method because the expected result of simulated data is known. Running the detection methods on the simulated data and analyzing the comparison between the running results and the expected results provide a good way to evaluate these detection methods. In addition, simulated data has very little data generation cost. Therefore, simulated data has turned out to be an important tool for data analysis. To better evaluate the performance of epistasis detection methods to detect SNP interactions in real biological SNP data, simulated datasets are critical.

In simulated data, a penetrance function, or a penetrance table, is commonly used to describe the epistatic relationship between SNPs. The penetrance table contains the probability of a particular combination of alleles and their expression phenotype. This probability is usually expressed as a penetrance value $P(D\left|g_{i}\right.)$ which is the probability of being affected given the genotype $g_{i}$ of the sample. We calculate the epistasis model in the simulated data, that is, we calculate the values of a penetrance table.

According to whether there is a marginal effect of a single SNP in the epistatic model, the models are divided into two types: the epistatic model with marginal effect (eME model) and the epistatic model without marginal effect (eNME model). The SNP epistatic model with marginal effect means that one or more SNPs in this is model have marginal effects, but the combined epistatic effect of all SNPs is stronger. To facilitate the calculation of this type of model, the penetrance value for each genotype combination is defined as a function of one or more variables, and each variable typically represents a statistical parameter of the interaction. Typically, the penetrance of a marginal effect epistatic model can be constrained to specific expressions for the baseline penetrance α and relative penetrance f [1,6,7]:

(1) $$P(D|g_{i})=F(\alpha ,f)$$

In this type of model, given the prevalence P(D) (the probability of a population affected by the epistasis model) and the heritability $h^{2}$ (the variation in phenotypes affected by epistatic models of SNP interactions) of the model where their formulas are as follows:

(2) $$P(D)=\sum_{{}_{i}}^{3^{K}}P(D|g_{i})P(g_{i})$$

(3) $$h^{2}=\frac{\sum_{{}_{i}}^{3^{K}}{\left(P(D|g_{i})-P(D)\right)}^{2}P(g_{i})}{P(D)(1-P(D))}$$

then both the values of α and f can be determined. Thus, the penetrance value of the combined genotype can be determined, and a specific epistatic model to be solved can be obtained. Under the constraints of prevalence and heritability, EpiSIM [1] solves α and f to obtain the eME model by solving equations, and at the same time, EpiSIM generates simulated data with linkage disequilibrium patterns and haplotype blocks using the Markov Chain process. This calculation method brings a lot of computational burdens when calculating high-order models. Toxo [8] calculates an eME model by specifying either prevalence or heritability and maximizing the other. In this way, it reduces the computational burden and can calculate high-order eME models, but Toxo does not have its own data generation method and needs to use other simulation software to generate simulated data. EpiGEN [9] also uses a similar parameter baseline risk to generate the model and it can generate both categorical and quantitative phenotypes, but it generates simulated data also with the help of other simulators.

The epistatic model without marginal effects means that a single SNP has no main effect, but the combination of the several specific SNPs has a strong epistatic effect [10,11]. The penetrance of the epistatic model without marginal effect has no obvious mathematical law, so it is impossible to constrain the epistatic models without marginal effects with functional expressions. Therefore, an epistatic model without marginal effect needs to calculate the penetrance value corresponding to the genotype separately, and make it satisfy the situation that the individual marginal effect of each SNP is equal to 0, which brings a heavy computational burden to the calculation of the model. GAMETES [12] is an epistasis simulator, which uses a random architecture to generate a pure and strict penetrance table of epistasis models without marginal effects, and the prevalence and heritability of the models to be generated are specified by users. EpiSIM [1] searches the value of penetrance with fixed steps in the range of 0 to 1 under the constraints of prevalence and heritability to calculate the penetrance table. Although EpiSIM uses an exhaustive search method to calculate the 2-order eNME model, it also brings a heavy computational burden.

In this study, we focus on the calculation of the epistatic model without marginal effects and propose a new simulation method called EpiReSIM. When calculating an eNME model, the simulator innovatively converts the calculation of the SNP epistasis model into the problem of solving the underdetermined equation by using the feature that the prevalence of the model is equal to the marginal penetrance. EpiReSIM divides the calculation of epistasis models displaying no marginal effects into two cases. In the first case, the calculation of the epistasis model displaying no marginal effects is a linear under-determined system of the equations-solving problem under only the prevalence constraint. In this case, we use the method of Complete Orthogonal Decomposition method to calculate this equation set. In the second case, when both prevalence and heritability are constrained simultaneously, we transform the computation of the epistasis model displaying no marginal effects into a nonlinear under-determined system of equations-solving problem, which is solved by the simulator using Newton’s method to calculate the penetrance value of the epistasis models displaying no marginal effects. Through the calculation of these two cases, EpiReSIM was able to compute penetrance tables for high-order models. Then, the simulation method also provides its own data generation, which uses a resampling method to generate samples of the simulated data and generates a label for each sample of simulated data based on the calculated penetrance table.

## 2. Materials and Methods

### 2.1. Genetics and Modeling

In order to clearly describe the effect of different genotypes in SNP interactions, we use the penetrance table to describe the epistatic model. A penetrance table includes the genotypes of the SNP locus and the penetrance values corresponding to these genotypes. As SNP is a single locus in the DNA sequence, it refers to the polymorphism of the DNA sequence caused by a single deoxyribonucleotide variation in the genome. Most of the SNPs that are characterized are as biallelic, meaning that only two alleles (*A* or a) are observed in a population. Under the condition of satisfying Hardy-Weinberg Equilibrium (HWE) [13], the genotype of the SNP locus can be calculated as:

(4) $$P\left(AA\right)={\left(1-p\right)}^{2},\;P\left(Aa\right)=2p\left(1-p\right)\;,\;P\left(aa\right)=p^{2}$$

where p is the minor allele frequencies (MAFs) of the minor allele ‘a’. Therefore, in a K-order epistatic model, given the MAFs of the $SNP_{k}$ locus as $MAF_{k}$ where $k\in \left\{1,2,\cdot \cdot \cdot ,K\right\}$, the genotype frequency of the SNP locus (AA,Aa,aa) can be calculated as:

(5) $$q_{k}=({\left(1-MAF_{k}\right)}^{2},2MAF_{k}(1-MAF_{k}),MAF_{k}{}^{2})$$

Suppose that the SNPs of K-order epistatic model are in linkage equilibrium, that is, these SNPs are independent of each other [7]. For a given $MAF_{k}$ of $SNP_{k}$, the combined genotype frequency $P(g_{i})$, $i\in \left\{1,2,\cdot \cdot \cdot ,3^{K}\right\}$ is constant and is the product of the corresponding genotype frequencies for each SNP.

Table 1 is an example showing the penetrance table for an epistatic model without marginal effects when $MAF_{1}=MAF_{2}=0.2,\;h^{2}=0.4,P(D)=0.64$ [14,15], and the penetrance values in the table are all in the range of $\left[0,1\right]$. Since there is no marginal effect, this type of model satisfies the inherent relations that the marginal penetrance values are equal to the prevalence value [11]. Given an SNP epistatic model and a genotype for that epistatic model, such as AA, the probability of all affected individuals with genotype AA in the population is called the marginal penetrance of AA on the SNP epistatic model, usually denoted by P(D|AA). Its calculation formula is:

(6) $$P(D|AA)=\sum_{j}\left(P(D|g_{AAj})P_{gj}\right)$$

If a 2-order SNP epistatic model with no marginal effect is given, then the marginal penetrance of the model satisfies:

(7) P(D|AA)=P(D|Aa)=P(D|aa)=P(D|BB)=P(D|Bb)=P(D|bb)=P(D)

In the case of Table 1, if an individual has genotype *AA* and the genotype of the SNP2 is not considered, then the marginal penetrance of the genotypes AA is:

(8) P(D|AA)=0.64×0.4865+0.32×0.9473+0.04×0.6401=0.64

which is equal to the prevalence P(D) of the epistatic model. If it has genotype *Aa*, the marginal penetrance of the genotypes Aa is:

(9) P(D|Aa)=0.64×0.9601+0.32×0.0042+0.04×0.6065=0.64

and for genotype aa, the marginal penetrance is:

(10) P(D|aa)=0.64×0.5377+0.32×0.8113+0.04×0.9089=0.64

which are also equal to the prevalence P(D) of the epistatic model. Similar computations give the same value of 0.64 for the three values of the marginal penetrance associated with SNP2.

### 2.2. Calculating of Epistatic Model

Since the penetrance in the eNME model has no rules, the penetrance values cannot be constrained to the form of a function expression. Thus, $3^{K}$ penetrance values of the K-order model should be calculated separately and the condition that these marginal penetrances are equal to the prevalence values should be met. In this study, we regard the penetrance to be solved as an unknown, and the calculation of the prevalence table becomes the problem of solving the system of equations. In this case, the penetrance function to be solved can be written in Table 2. In this paper, we divide the calculation of the model into two ways: using prevalence and using prevalence and heritability.

When using the prevalence to calculate the eNME model, under the constraints of marginal penetrance and prevalence, the calculation formulas are linear equations with $3^{K}$ penetrance unknowns where K is the order of the eNME model. After analysis, there are $3^{K}$ penetrance values to be calculated, while the number of equations is only (3×K+1) and the number of equations is much smaller than the number of unknowns. Therefore, in this case, we simplify the computation of the eNME model to the problem of finding a solution of the under-determined linear system, where the penetrance values in the eNME model are unknown in the system of equations. We formulate the required problem as:

(11) $$Ax=bA\in {\mathbb{R}}^{m\times n},b\in {\mathbb{R}}^{m}$$

where m<n and m=3×K+1, $n=3^{K}$. Notice that such as the linear under-determined system has an infinity of solutions. On the one hand, we look for a minimum 2-norm solution because the solution of the minimum 2-norm is unique. On the other hand, what is more, important is that the value in the penetrance table for the eNME model that we need to solve for is basically a probability value, ranging from 0 to 1. The minimum 2-norm finds a solution that is closer to the origin of the coordinate axis. EpiReSIM introduces minimizing 2-norm into the process of solving the eNME model to help EpiReSIM find a solution that is more consistent with the constraint conditions. In that case, EpiReSIM tries to find a minimum 2-norm solution.

Here it uses the algorithm of Complete Orthogonal Decomposition (COD) [16] to the linear under-determined system. Similar to QR decomposition and Singular Value Decomposition (SVD) method, Complete Orthogonal Decomposition is also a method to solve the matrix equation. Through analysis, the matrix in the process of solving the penetrance table of eNME model is a non-full rank matrix, that is: $rank\left(A\right)<m$. Therefore, we use the method of COD to calculate under-determined linear equations, which is an alternative to the SVD and it is usually faster and about as accurate. The COD decomposition of the matrix A is a mathematical operation that generates three matrices Q, Z and $R_{11}$. It can be written as:

(12) $$AP=Q\left(\begin{array}{cc} R_{11} & 0\\ 0 & 0 \end{array}\right)Z^{T}$$

where the matrices of Q and Z are orthogonal which means the inner product of any two columns of Q is zero, and the inner product of a column with itself is one ($QQ^{T}=E$). $R_{11}$ is an upper triangular matrix which means that any element under the main diagonal is zero. P is a permutation matrix. With this computational strategy, EpiReSIM is better able to solve for the solution of the eNME model.

When using both prevalence and heritability to calculate the eNME model, the system of equations to be solved becomes an under-determined nonlinear equation because the formula of heritability is a quadratic function. Here, EpiReSIM uses Newton’s method [17,18,19] to solve the target problem.

When using the Newton’s method, we organize the performance of the objective function as:

(13) $$\left\{\begin{array}{c} f_{1}(x_{1},x_{2},\cdot \cdot \cdot x_{3^{K}})=0\\ f_{2}(x_{1},x_{2},\cdot \cdot \cdot x_{3^{K}})=0\\ \ldots \\ f_{\left(3*K+2\right)}(x_{1},x_{2},\cdot \cdot \cdot x_{3^{K}})=0 \end{array}\right.$$

where $x_{1},x_{2},\cdot \cdot \cdot x_{3^{K}}$ are the penetrance values of the eNME model that needs to be solved, K is the order of the eNME model, $F(x)={\left(f_{1},f_{2},\cdot \cdot \cdot f_{3*K+2}\right)}^{T}$ are the formula for calculating marginal penetrance, prevalence, and heritability. We solve for the value of penetrance according to these formulas. EpiReSIM uses the classical iterative method, Newton’s method, for solving (13), which generates an iterate sequence $x_{i+1}$ of the form:

(14) $$x_{i+1}=x_{i}-J{\left(x_{i}\right)}^{-1}F(x_{i}),i=0,1,2,\cdot \cdot \cdot ,$$

where $J(x_{i})$ is the Jacobian matrix of F(x). For the convenience of calculation, we convert the form of the formula into:

(15) $$\left\{\begin{array}{c} x_{i+1}=x_{i}+\Delta x_{i}\\ J(x_{i})\Delta x_{i}+F(x_{i})=0 \end{array}\right.$$

Therefore, given $x_{0}$ as a zero vector, the nonlinear system of equations can be solved.

### 2.3. Resampling to Generate Samples

To generate samples of the simulated data, EpiReSIM requires a real biological SNP dataset. Here we use a SNP dataset of Type 1 Diabetes (T1D). This dataset is processed as a matrix, where each row represents a sample and each column represents a SNP. The elements in the matrix are the genotypes $g_{i}$, and EpiReSIM uses (1, 2, 3) to denote the genotypes $\left(AA,\;Aa,\;aa\right)$ of the samples. In the matrix of real biological data, each sample has a lot of the samples and each sample has a label. According to the label, these samples are divided into control group and case group, where the control group is labeled by 0 and the case group is labeled by 1.

The generation process of these samples is as follows. First, a certain number of consecutive SNPs are taken out at random positions in the real biological data, and this number is the value of the SNPs in the simulated data that the user needs. Second, in the SNP data generated above, the samples labeled as 1 are removed to reduce the influence of the original epistasis model in the real biological data when the epistasis model is later embedded. Then, these data processed above are used to generate SNP simulated data by a resampling method. To generate a sample of simulated data, each sample of these preprocessed data is randomly segmented according to the column, and each segment is a random number of SNPs. Therefore, these preprocessed data are divided into random groups where each group contains several SNP fragments. A sample of simulated data is generated by randomly selecting a segment in each group and splicing these segments in turn. Suppose a real biological dataset has 200 samples (rows), and each sample is divided into 50 segments. The simulated data for a sample is generated by randomly selecting a segment from the first group, randomly selecting a segment from the second group, randomly selecting a segment from the third group, and so on until all 50 groups have been selected. Then the 50 selected segments are spliced together in turn to generate a sample of simulated data. Thus, the simulated data retains the basic patterns of linkage disequilibrium, and MAFs as that observed in the real dataset [20].

### 2.4. Generating the Labels of Samples

To embed the epistasis model into the simulated data, it generates a label for each sample and divides these samples into case group and control group according to the values in the calculated penetrance table. The sample label is determined by the genotype of the locus of the SNP epistasis model $g_{i}$ and the penetrance value of this genotype $P(D|g_{i})$. The probability that a sample is labelled as a case is the penetrance value $P(D|g_{i})$ in epistasis model if the model affects it. In the final outputs, it can generate a target number of case group samples and control group samples according to the needs of users.

## 3. Results

EpiReSIM has he following main properties: preservation of realistic MAFs, accuracy epistasis model embedding and acceptable generating time. To evaluate the applicability of these properties, we conduct experimental verifications from the following aspects. First, we compare the MAFs between the simulated dataset and the real dataset, validating the ability of EpiReSIM to preserve the MAF of the real dataset. Second, after eight epistasis models are calculated and embedded in the simulated dataset, we use some SNP epistasis detection methods to identify these embedded eNME models, and experimental results demonstrate the accuracy of EpiReSIM in embedding models. Third, comparing EpiReSIM with other simulation software, we record the running time of generating different kinds of datasets, illustrating the usability of generating large datasets. Fourthly, we have compared EpiReSIM to other existing simulators to demonstrate its flexibility and practicality.

### 3.1. Preservation of Realistic MAFs

**Figure 1:**
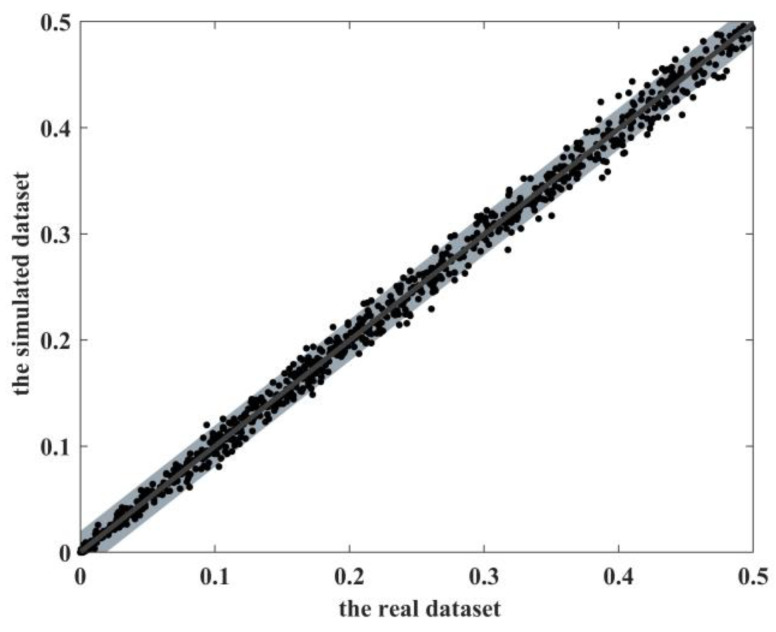
The comparisons of MAFs between the simulated dataset and the real dataset.

To analyze the ability of EpiReSIM to preserve the realistic MAF, we use this simulation method to generate a dataset with 1000 SNPs and 1000 samples and calculate the MAF of the corresponding loci in the real biological dataset and the MAF in the simulated dataset respectively to analyze their distribution. Figure 1 shows the comparisons of MAFs between the simulated dataset and the real biological dataset. The *X*-axis in the figure is the MAF of the real dataset, and the *Y*-axis is the MAF of the simulated data. Each point in the figure is the MAF value of the same SNP loci corresponding to the simulated dataset and the real biological dataset. As shown by the dark area in the Figure 1, we calculate the 95% confidence interval of MAF observed by each SNP in the simulated sample. It can be seen from Figure 1 that the MAF in the simulated data is not much different from the real biological dataset, which not only preserves the characteristics in the real dataset, but also has a certain specificity.

### 3.2. Accuracy Epistasis Model Embedding

To verify the accuracy of the epistasis model embedded in the simulated data generated by the simulator, we use the simulator to calculate eight eNME models using the only constraint of prevalence. In the eight eNME models, Model 1 to Model 4 are 2-order eNME models, Model 5 and Model 6 are 3-order eNME models and Model 7 and Model 8 are 4-order eNME models Then, we generate the simulated datasets with different sample sizes, 1000 samples, 2000 samples and 4000 samples (2000 case samples and 2000 control samples) and each of which contains 1000 SNPs. For each setting, 100 data sets are generated. Table 3 is the details of these eNME models. Then these eNME models are embedded into different simulated datasets respectively.

After generating the simulated datasets, we use a SNP epistasis detection method: MDR [21] to detect the eNME model in the simulated datasets. The software package for MDR is publicly available and it claims to be suitable for the detection of SNP epistasis interaction. In this experiment, the parameters of the epistasis detection method are set to default values. These detection results of MDR are shown in Table 4. Power (detection rate) is the probability of successful detection. We analyze the comparison between the running results and the expected results. It can be seen from the detection results that under certain combinations of parameters, the embedded model has a probability of being detected.

**Figure 2:**
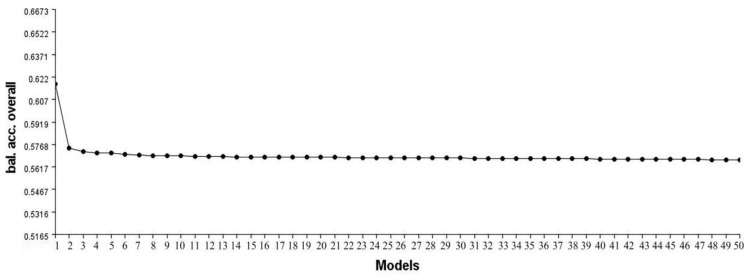
The detected result of MDR on Model 5.

It also shows the detection result of MDR on Model 5 in the simulated data, and the detection results are shown in Figure 2. *Y*-axis is the balanced accuracy in whole datasets and *X*-axis is the number of the top 50 SNP combinations in the detection result. These points in this figure represent the accuracy values corresponding to the top 50 SNP combinations that can be detected. The SNP loci (193, 339, 962) embedded in the simulated data are the first to be found. It can be seen this embedded eNME model has the highest accuracy value in the detected result, while the combinations of non-model SNPs represented by other points in the simulated data are randomly combined and have similar accuracy values. Figure 2 signifies that the model embedded by our method is accurate and not affected by other SNP combinations.

To determine the range of heritability, prevalence and MAFs values set by this simulation method when finding a solution for each of the evaluated order, we conducted the experiments and presented the experimental results in Supplementary Tables S1–S22. By analyzing the experimental results, we give the range of parameters calculated for the model of this simulation method as: First, our suggestion is to use the only constraint of prevalence to calculate the eNME model because it has a greater successful rate of calculating the model than using both prevalence and heritability constraints. Secondly, the recommended parameters for the calculation of the eNME model are: the range of the prevalence of the 2-order and 2-order models is 0 to 0.3, and the range of heritability is 0 to 0.3. The recommended prevalence of the 4-order model is 0 to 0.2, and the heritability is 0 to 0.2. Of course, neither of these two parameters can be taken as 0. If you want to obtain a higher successful rate of calculation, you can constrain each MAF to be within the range of 0.05 to 0.3.

### 3.3. Acceptable Generating Time

**Figure 3:**
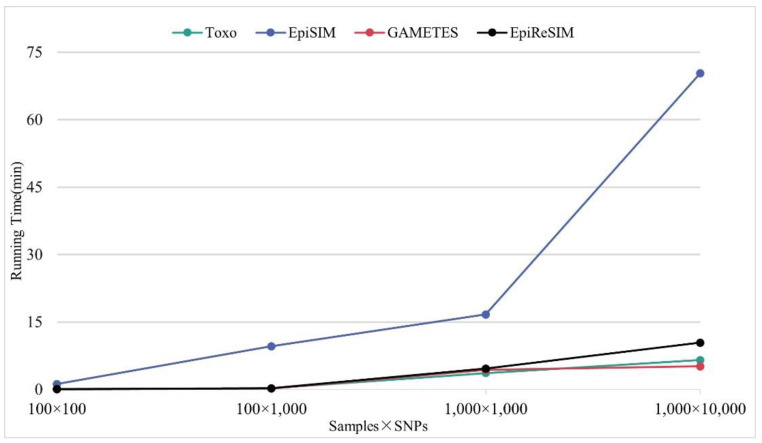
Running time of different simulators.

To demonstrate that the EpiReSIM can generate the simulated data within an acceptable running time, we set different numbers of SNPs and sample sizes in the simulation method to generate the simulated data. At the same time, we recorded the corresponding running times and compared them with other simulation software. To avoid outliers, 100 data are generated for each data set and all the generation processes are repeated 30 times. Figure 3 shows the results of comparing the running times under different settings. *Y*-axis is the minutes of generating the simulated data. *X*-axis is the size of the different data sets, expressed as the product of two numbers where the former is the number of samples and the latter is the size of the SNPs. The results show that the running time of EpiReSIM increases linearly with the number of samples and SNPs. Thus, EpiReSIM can generate relatively large-scale data set in an acceptable run time. Although the running time of EpiReSIM is not the fastest on the last dataset, its running time is only slightly lower than that of the fastest method.

Then, we set different parameter combinations to calculate the eNME model using these two strategies and recorded the calculation time. We calculated the model with different parameters 100 times and recorded the average calculation time. Table 5 shows the calculation times for generating different eNME models. By analyzing the experimental results, it can be seen that when EpiReSIM is calculated at the same order, the running time is close and the calculation time with two constraints is slightly greater than that with one constraint. Therefore, the proposed simulation method does not have the problem of model preference.

### 3.4. Comparison with Existing Simulators

We select three representative simulators for comparison with EpiReSIM, namely EpiSIM [1], GAMETES [12], and Toxo [8], all of which use penetrance tables to represent epistasis models. The comparison results are shown in Table 6. Compared with other simulators, EpiReSIM has several advantages and outstanding functions. First, compared with GAMETES and Toxo, EpiReSIM uses a resampling method to generate simulated data and can well preserve the biological properties of the MAFs of the real biological SNP dataset, which can make the simulated data closer to the real biological dataset. Second, compared with EpiSIM, EpiReSIM shows better capability in model calculation. EpiReSIM not only shows a better success rate in the calculation of the eNME model, but also supports the calculation of high-order eNME models. Therefore, it can be argued that EpiReSIM is a convenient and effective alternative method to current simulation software.

## 4. Conclusions

The main contribution of this work is to propose a simulator, EpiReSIM, which can calculate the penetrance table of high-order eNME models and generate simulated data by resampling method. EpiReSIM provides two calculation methods, that is users can calculate the model by using the constraints on prevalence or using the constraints of prevalence and heritability. The validity of the simulated data generated by EpiReSIM is verified by different SNP interaction recognition methods. At the same time, the simulator is open source, which facilitates the study of SNP interaction methods.

This simulation method, however, also has its own limitations and there are several areas that need to be enhanced. First, although EpiReSIM can calculate high-order models without marginal effects, there are very few cases where the heritability of the calculated models is relatively low, which puts forward higher requirements for SNP interaction identification methods. Second, the current version of EpiReSIM does not consider interactions between environmental and genetic factors. We will focus on improving the usability of EpiReSIM in the following two areas: including the influence of environmental factors when calculating the eNME model, and optimizing the data generation method of resampling.

## Figures and Tables

**Table 1:** The penetrance table of a 2-order eNME model.

		SNP1			Marginal Penetrance
	Genotype	AA(0.64)	Aa(0.32)	aa(0.04)	
SNP2	BB(0.64)	0.4865	0.9601	0.5377	0.64
	Bb(0.32)	0.9473	0.0042	0.8113	0.64
	bb(0.04)	0.6401	0.6065	0.9089	0.64
	Marginal penetrance	0.64	0.64	0.64	*P* (*D*) = 0.64

**Table 2:** The penetrance function of a 2-order eNME model.

		SNP1			Marginal Penetrance
	Genotype	AA	Aa	aa	
SNP2	BB	$x_{1}$	$x_{2}$	$x_{3}$	*P* (*D*|*BB*)
	Bb	$x_{4}$	$x_{5}$	$x_{6}$	*P* (*D*|*Bb*)
	bb	$x_{7}$	$x_{8}$	$x_{9}$	*P* (*D*|*bb*)
	Marginal penetrance	*P* (*D*|*AA*)	*P* (*D*|*Aa*)	*P* (*D*|*aa*)	*P* (*D*)

**Table 3:** The details of these eNME models.

	Order	*P* (*D*)	SNP Loci
Model 1	2-order	0.1	825, 511
Model 2	2-order	0.2	197, 52
Model 3	2-order	0.3	687, 74
Model 4	2-order	0.4	23, 696
Model 5	3-order	0.3	962, 193, 339
Model 6	3-order	0.4	461, 755, 428
Model 7	4-order	0.1	176, 76, 439, 465
Model 8	4-order	0.2	497, 46, 362, 123

**Table 4:** The results of the MDR on the simulated data.

Model	Results	Power		
		1000 Samples	2000 Samples	4000 Samples
Model 1	511, 825	0.15	0.80	1.00
Model 2	52, 197	0.20	0.90	1.00
Model 3	74, 687	0.70	1.00	1.00
Model 4	23, 696	1.00	1.00	1.00
Model 5	193, 339, 962	0.45	0.95	1.00
Model 6	428, 461, 755	0.25	0.80	1.00
Model 7	76, 176, 439, 465	0.75	1.00	1.00
Model 8	46, 123, 362, 497	0.80	1.00	1.00

**Table 5:** The running time with different models.

	*MAF*	*P* (*D*)	*Time* (s)	*P* (*D*)	*h* ^2^	*Time* (s)
2-order	0.1, 0.4	0.1	3.4554	0.2	0.1	3.6100
	0.2, 0.3	0.1	3.0771	0.2	0.1	3.4776
	0.1, 0.4	0.2	2.9695	0.1	0.05	3.2221
	0.2, 0.3	0.2	2.8789	0.1	0.05	3.4221
3-order	0.1, 0.4, 0.2	0.1	4.5305	0.2	0.05	5.9646
		0.2	4.0120	0.1	0.1	5.6743
4-order	0.1, 0.3, 0.2, 0.4	0.1	8.4997	0.1	0.05	13.3102
		0.15	8.9237	0.05	0.05	13.4144

**Table 6:** Comparison results of EpiReSIM with existing simulators.

	EpiSIM	GAMETES	Toxo	EpiReSIM
preserve MAF of real datasets	×	×	×	√
simulate high-order interactions	×	√	√	√
support MAF specification	√	√	√	√
not depend on other software	√	√	×	√

## Data Availability

The source code of EpiReSIM, usage instructions and all the models and code examples used within this paper, are available in the Github repository: https://github.com/CDMB-lab/EpiReSIM (accessed on 17 October 2022).

## Supplementary Materials

The following are available online at https://www.mdpi.com/article/10.3390/genes13122286/s1, Table S1: The frequency of successful generation of eNME model with only prevalence specified; Table S2: The frequency of successful generation of 2-order eNME model with both prevalence and heritability specified; Table S3: The frequency of successful generation of 3-order eNME model with both prevalence and heritability specified; Table S4: The frequency of successful generation of 4-order eNME model with both prevalence and heritability specified; Table S5: The detailed table of successful generation frequencies for 2-order eNME model with only prevalence specified; Table S6: The detailed table of successful generation frequencies for 2-order eNME model with $h^{2}=0.05$ Table S7: The detailed table of successful generation frequencies for 2-order eNME model with $h^{2}=0.1$; Table S8: The detailed table of successful generation frequencies for 2-order eNME model with $h^{2}=0.15$; Table S9: The detailed table of successful generation frequencies for 2-order eNME model with $h^{2}=0.2$; Table S10: The detailed table of successful generation frequencies for 2-order eNME model with $h^{2}=0.3$; Table S11: The detailed table of successful generation frequencies for 3-order eNME model with only prevalence specified; Table S12: The detailed table of successful generation frequencies for 3-order eNME model with $h^{2}=0.05$; Table S13: The detailed table of successful generation frequencies for 3-order eNME model with $h^{2}=0.1$; Table S14: The detailed table of successful generation frequencies for 3-order eNME model with $h^{2}=0.15$; Table S15: The detailed table of successful generation frequencies for 3-order eNME model with $h^{2}=0.2$; Table S16: The detailed table of successful generation frequencies for 3-order eNME model with $h^{2}=0.3$; Table S17: The detailed table of successful generation frequencies for 4-order eNME model with only prevalence specified; Table S18: The detailed table of successful generation frequencies for 4-order eNME model with $h^{2}=0.05$; Table S19: The detailed table of successful generation frequencies for 4-order eNME model with $h^{2}=0.1$; Table S20: The detailed table of successful generation frequencies for 4-order eNME model with $h^{2}=0.15$; Table S21: The detailed table of successful generation frequencies for 4-order eNME model with $h^{2}=0.2$; Table S22: The detailed table of successful generation frequencies for 4-order eNME model with $h^{2}=0.3$.
