# Supplementary material for: EpiReSIM: A Resampling Method of Epistatic Model without Marginal Effects Using Under-Determined System of Equations
Source: Genes (Basel). 2022 Dec 4;13(12):2286. doi: 10.3390/genes13122286 (PMC9777644; doi:10.3390/genes13122286)
Supplement: Supplementary file 1 [file genes-13-02286-s001.zip › genes-2006546-supplementary.pdf]

EpiReSIM: a resampling method of epistatic model without marginal effects using under-determined system of equations

- Supplementary file

Junliang Shang[1], Xinrui Cai[1], Tongdui Zhang[3], Yan Sun[1], Yuanyuan Zhang[2], Jin-Xing Liu[1], Boxin Guan[1, *]

1.Calculation of parameter range

We set different parameter combinations: the range of MAF is 0.1 to 0.5, the range of heritability is 0.05 to 0.3, and the range of prevalence is 0.1 to 0.5 and use two different strategies to calculate the model for different parameter combinations separately to determine the range of heritability, prevalence and MAFs values set by this simulation method when finding a solution for each of the evaluated order. For each combination of parameters, we calculated 200 times to reduce the error. The results of the experiment are as follows:

Table S1: The frequency of successful generation of eNME model with only prevalence specified.

Order \ $P(D)$	0.1	0.2	0.3	0.4	0.5
2-order	1.0000	1.0000	1.0000	1.0000	1.0000
3-order	1.0000	1.0000	1.0000	1.0000	1.0000
4-order	0.7347	0.5225	0.3653	0.2428	0.0837

Table S2: The frequency of successful generation of 2-order eNME model with both prevalence and heritability specified.

h^2 \ $P(D)$	0.1	0.2	0.3	0.4	0.5
0.05	1.0000	1.0000	1.0000	1.0000	1.0000
0.1	1.0000	1.0000	1.0000	1.0000	1.0000
0.15	0.9333	0.9333	0.9333	0.7580	0.7550
0.2	1.0000	1.0000	0.7590	0.1460	0.1190
0.3	0.7330	1.0000	0.1340	0.0330	0.0330

Table S3: The frequency of successful generation of 3-order eNME model with both prevalence and heritability specified.

h^2 \ $P(D)$	0.1	0.2	0.3	0.4	0.5
0.05	0.9269	0.6434	0.3164	0.2163	0.0489
0.1	0.9073	0.6400	0.3180	0.1337	0.0638
0.15	0.9041	0.6135	0.2919	0.0930	0.0014
0.2	0.9020	0.6097	0.2473	0.0023	0.0000
0.3	0.8721	0.6407	0.0684	0.0000	0.0000

Table S4: The frequency of successful generation of 4-order eNME model with both prevalence and heritability specified.

h^2 \ $P(D)$	0.1	0.2	0.3	0.4	0.5
0.05	0.9269	0.0016	0.0003	0.0000	0.0000
0.1	0.3758	0.0139	0.0000	0.0000	0.0000
0.15	0.3856	0.0339	0.0000	0.0000	0.0000
0.2	0.3911	0.0269	0.0000	0.0000	0.0000
0.3	0.3701	0.0328	0.0000	0.0000	0.0000

Table S5: The detailed table of successful generation frequencies for 2-order eNME model with only prevalence specified.

	$MAF_1 = 0.1$				
$P(D)$ / MAF_2	0.1	0.2	0.3	0.4	0.5
0.1	1.000	1.000	1.000	1.000	1.000
0.2	1.000	1.000	1.000	1.000	1.000
0.3	1.000	1.000	1.000	1.000	1.000
0.4	1.000	1.000	1.000	1.000	1.000
0.5	1.000	1.000	1.000	1.000	1.000
	$MAF_1 = 0.2$				
$P(D)$ / MAF_2	0.1	0.2	0.3	0.4	0.5
0.2	1.000	1.000	1.000	1.000	1.000
0.3	1.000	1.000	1.000	1.000	1.000
0.4	1.000	1.000	1.000	1.000	1.000
0.5	1.000	1.000	1.000	1.000	1.000
	$MAF_1 = 0.3$				
$P(D)$ / MAF_2	0.1	0.2	0.3	0.4	0.5
0.3	1.000	1.000	1.000	1.000	1.000
0.4	1.000	1.000	1.000	1.000	1.000
0.5	1.000	1.000	1.000	1.000	1.000
	$MAF_1 = 0.4$				
$P(D)$ / MAF_2	0.1	0.2	0.3	0.4	0.5
0.4	1.000	1.000	1.000	1.000	1.000
0.5	1.000	1.000	1.000	1.000	1.000
	$MAF_1 = 0.5$				
$P(D)$ / MAF_2	0.1	0.2	0.3	0.4	0.5
0.5	1.000	1.000	1.000	1.000	1.000

Table S6: The detailed table of successful generation frequencies for 2-order eNME model with $h^2 = 0.05$.

	$h^2 = 0.05, MAF_1 = 0.1$				
$P(D)$ / MAF_2	0.1	0.2	0.3	0.4	0.5
0.1	1.000	1.000	1.000	1.000	1.000
0.2	1.000	1.000	1.000	1.000	1.000
0.3	1.000	1.000	1.000	1.000	1.000
0.4	1.000	1.000	1.000	1.000	1.000
0.5	1.000	1.000	1.000	1.000	1.000
	$h^2 = 0.05, MAF_1 = 0.2$				
$P(D)$ / MAF_2	0.1	0.2	0.3	0.4	0.5
0.2	1.000	1.000	1.000	1.000	1.000
0.3	1.000	1.000	1.000	1.000	1.000
0.4	1.000	1.000	1.000	1.000	1.000
0.5	1.000	1.000	1.000	1.000	1.000
	$h^2 = 0.05, MAF_1 = 0.3$				
$P(D)$ / MAF_2	0.1	0.2	0.3	0.4	0.5
0.3	1.000	1.000	1.000	1.000	1.000
0.4	1.000	1.000	1.000	1.000	1.000
0.5	1.000	1.000	1.000	1.000	1.000
	$h^2 = 0.05, MAF_1 = 0.4$				
$P(D)$ / MAF_2	0.1	0.2	0.3	0.4	0.5
0.4	1.000	1.000	1.000	1.000	1.000
0.5	1.000	1.000	1.000	1.000	1.000
	$h^2 = 0.05, MAF_1 = 0.5$				
$P(D)$ / MAF_2	0.1	0.2	0.3	0.4	0.5
0.5	1.000	1.000	1.000	1.000	1.000

Table S7: The detailed table of successful generation frequencies for 2-order eNME model with $h^2 = 0.1$.

	$h^2 = 0.1, MAF_1 = 0.1$				
MAF_2 \ $P(D)$	0.1	0.2	0.3	0.4	0.5
0.1	1.000	1.000	1.000	1.000	1.000
0.2	1.000	1.000	1.000	1.000	1.000
0.3	1.000	1.000	1.000	1.000	1.000
0.4	1.000	1.000	1.000	1.000	1.000
0.5	1.000	1.000	1.000	1.000	1.000
	$h^2 = 0.1, MAF_1 = 0.2$				
MAF_2 \ $P(D)$	0.1	0.2	0.3	0.4	0.5
0.2	1.000	1.000	1.000	1.000	1.000
0.3	1.000	1.000	1.000	1.000	1.000
0.4	1.000	1.000	1.000	1.000	1.000
0.5	1.000	1.000	1.000	1.000	1.000
	$h^2 = 0.1, MAF_1 = 0.3$				
MAF_2 \ $P(D)$	0.1	0.2	0.3	0.4	0.5
0.3	1.000	1.000	1.000	1.000	1.000
0.4	1.000	1.000	1.000	1.000	1.000
0.5	1.000	1.000	1.000	1.000	1.000
	$h^2 = 0.1, MAF_1 = 0.4$				
MAF_2 \ $P(D)$	0.1	0.2	0.3	0.4	0.5
0.4	1.000	1.000	1.000	1.000	1.000
0.5	1.000	1.000	1.000	1.000	1.000
	$h^2 = 0.1, MAF_1 = 0.5$				
MAF_2 \ $P(D)$	0.1	0.2	0.3	0.4	0.5
0.5	1.000	1.000	1.000	1.000	1.000

Table S8: The detailed table of successful generation frequencies for 2-order eNME model with $h^2 = 0.15$.

	$h^2 = 0.15, MAF_1 = 0.1$				
MAF_2 \ $P(D)$	0.1	0.2	0.3	0.4	0.5
0.1	1.000	1.000	1.000	1.000	1.000
0.2	1.000	1.000	1.000	1.000	1.000
0.3	1.000	1.000	1.000	1.000	1.000
0.4	1.000	1.000	1.000	1.000	1.000
0.5	1.000	1.000	1.000	1.000	1.000
	$h^2 = 0.15 MAF_1 = 0.2$				
MAF_2 \ $P(D)$	0.1	0.2	0.3	0.4	0.5
0.2	1.000	1.000	1.000	1.000	1.000
0.3	1.000	1.000	1.000	1.000	1.000
0.4	1.000	1.000	1.000	1.000	1.000
0.5	1.000	1.000	1.000	1.000	1.000
	$h^2 = 0.15 MAF_1 = 0.3$				
MAF_2 \ $P(D)$	0.1	0.2	0.3	0.4	0.5
0.3	1.000	1.000	1.000	1.000	0.990
0.4	1.000	1.000	1.000	0.260	0.000
0.5	1.000	1.000	1.000	0.000	0.000
	$h^2 = 0.15, MAF_1 = 0.4$				
MAF_2 \ $P(D)$	0.1	0.2	0.3	0.4	0.5
0.4	1.000	1.000	1.000	0.990	0.930
0.5	1.000	1.000	1.000	0.120	0.020
	$h^2 = 0.15, MAF_1 = 0.5$				
MAF_2 \ $P(D)$	0.1	0.2	0.3	0.4	0.5
0.5	0.000	0.000	0.000	0.000	0.390

Table S9: The detailed table of successful generation frequencies for 2-order eNME model with $h^2 = 0.2$.

	$h^2 = 0.2, MAF_1 = 0.1$				
$P(D)$ / MAF_2	0.1	0.2	0.3	0.4	0.5
0.1	1.000	1.000	0.000	0.000	0.000
0.2	1.000	1.000	0.000	0.000	0.490
0.3	1.000	1.000	1.000	0.005	0.000
0.4	1.000	1.000	1.000	0.000	0.000
0.5	1.000	1.000	0.035	0.000	0.000
	$h^2 = 0.2, MAF_1 = 0.2$				
$P(D)$ / MAF_2	0.1	0.2	0.3	0.4	0.5
0.2	1.000	1.000	1.000	0.280	0.990
0.3	1.000	1.000	1.000	0.000	0.000
0.4	1.000	1.000	1.000	0.000	0.000
0.5	1.000	1.000	0.350	0.000	0.000
	$h^2 = 0.2, MAF_1 = 0.3$				
$P(D)$ / MAF_2	0.1	0.2	0.3	0.4	0.5
0.3	1.000	1.000	1.000	0.915	0.295
0.4	1.000	1.000	1.000	0.000	0.000
0.5	1.000	1.000	1.000	0.000	0.000
	$h^2 = 0.2, MAF_1 = 0.4$				
$P(D)$ / MAF_2	0.1	0.2	0.3	0.4	0.5
0.4	1.000	1.000	1.000	0.860	0.000
0.5	1.000	1.000	1.000	0.015	0.000
	$h^2 = 0.2, MAF_1 = 0.5$				
$P(D)$ / MAF_2	0.1	0.2	0.3	0.4	0.5
0.5	1.000	1.000	1.000	0.115	0.010

Table S10: The detailed table of successful generation frequencies for 2-order eNME model with $h^2 = 0.3$.

	$h^2 = 0.3, MAF_1 = 0.1$				
$P(D)$ / MAF_2	0.1	0.2	0.3	0.4	0.5
0.1	1.000	1.000	0.000	0.000	0.000
0.2	1.000	1.000	0.000	0.000	0.000
0.3	1.000	1.000	0.000	0.000	0.000
0.4	1.000	1.000	0.000	0.000	0.000
0.5	1.000	1.000	0.000	0.000	0.000
	$h^2 = 0.3, MAF_1 = 0.2$				
$P(D)$ / MAF_2	0.1	0.2	0.3	0.4	0.5
0.2	0.000	1.000	0.000	0.000	0.000
0.3	0.000	1.000	0.000	0.000	0.000
0.4	0.000	1.000	0.000	0.000	0.000
0.5	0.000	1.000	0.000	0.000	0.000
	$h^2 = 0.3, MAF_1 = 0.3$				
$P(D)$ / MAF_2	0.1	0.2	0.3	0.4	0.5
0.3	1.000	1.000	0.000	0.000	0.000
0.4	1.000	1.000	0.000	0.000	0.000
0.5	1.000	1.000	0.000	0.000	0.000
	$h^2 = 0.3, MAF_1 = 0.4$				
$P(D)$ / MAF_2	0.1	0.2	0.3	0.4	0.5
0.4	1.000	1.000	0.955	0.000	0.000
0.5	1.000	1.000	0.050	0.000	0.000
	$h^2 = 0.3, MAF_1 = 0.5$				
$P(D)$ / MAF_2	0.1	0.2	0.3	0.4	0.5
0.5	1.000	1.000	1.000	0.500	0.050

Table S11: The detailed table of successful generation frequencies for 3-order eNME model with only prevalence specified.

	$MAF_1 = 0.1, MAF_2 = 0.1$				
$P(D)$ / MAF_3	0.1	0.2	0.3	0.4	0.5
0.1	1.000	1.000	1.000	1.000	1.000
0.2	1.000	1.000	1.000	1.000	1.000
0.3	1.000	1.000	1.000	1.000	1.000
0.4	1.000	1.000	1.000	1.000	1.000
0.5	1.000	1.000	1.000	1.000	1.000

	$MAF_1 = 0.1, MAF_2 = 0.2$				
$P(D)$ / MAF_3	0.1	0.2	0.3	0.4	0.5
0.2	1.000	1.000	1.000	1.000	1.000
0.3	1.000	1.000	1.000	1.000	1.000
0.4	1.000	1.000	1.000	1.000	1.000
0.5	1.000	1.000	1.000	1.000	1.000

	$MAF_1 = 0.1, MAF_2 = 0.3$				
$P(D)$ / MAF_3	0.1	0.2	0.3	0.4	0.5
0.3	1.000	1.000	1.000	1.000	1.000
0.4	1.000	1.000	1.000	1.000	1.000
0.5	1.000	1.000	1.000	1.000	1.000

	$MAF_1 = 0.1, MAF_2 = 0.4$				
$P(D)$ / MAF_3	0.1	0.2	0.3	0.4	0.5
0.4	1.000	1.000	1.000	1.000	1.000
0.5	1.000	1.000	1.000	1.000	1.000

	$MAF_1 = 0.1, MAF_2 = 0.5$				
$P(D)$ / MAF_3	0.1	0.2	0.3	0.4	0.5
0.5	1.000	1.000	1.000	1.000	1.000

	$MAF_1 = 0.2, MAF_2 = 0.2$				
$P(D)$ / MAF_3	0.1	0.2	0.3	0.4	0.5
0.2	1.000	1.000	1.000	1.000	1.000
0.3	1.000	1.000	1.000	1.000	1.000
0.4	1.000	1.000	1.000	1.000	1.000
0.5	1.000	1.000	1.000	1.000	1.000

	$MAF_1 = 0.2, MAF_2 = 0.3$				
$P(D)$ / MAF_3	0.1	0.2	0.3	0.4	0.5
0.3	1.000	1.000	1.000	1.000	1.000
0.4	1.000	1.000	1.000	1.000	1.000
0.5	1.000	1.000	1.000	1.000	1.000

	$MAF_1 = 0.2, MAF_2 = 0.4$				
$P(D)$ / MAF_3	0.1	0.2	0.3	0.4	0.5
0.4	1.000	1.000	1.000	1.000	1.000
0.5	1.000	1.000	1.000	1.000	1.000

	$MAF_1 = 0.2, MAF_2 = 0.5$				
$P(D)$ / MAF_3	0.1	0.2	0.3	0.4	0.5
0.5	1.000	1.000	1.000	1.000	1.000

	$MAF_1 = 0.3, MAF_2 = 0.3$				
$P(D)$ / MAF_3	0.1	0.2	0.3	0.4	0.5
0.3	1.000	1.000	1.000	1.000	1.000
0.4	1.000	1.000	1.000	1.000	1.000
0.5	1.000	1.000	1.000	1.000	1.000

	$MAF_1 = 0.3, MAF_2 = 0.4$				
$P(D)$ / MAF_3	0.1	0.2	0.3	0.4	0.5
0.4	1.000	1.000	1.000	1.000	1.000
0.5	1.000	1.000	1.000	1.000	1.000

	$MAF_1 = 0.3, MAF_2 = 0.5$				
$P(D)$ / MAF_3	0.1	0.2	0.3	0.4	0.5
0.5	1.000	1.000	1.000	1.000	1.000

	$MAF_1 = 0.4, MAF_2 = 0.4$				
$P(D)$ / MAF_3	0.1	0.2	0.3	0.4	0.5
0.4	1.000	1.000	1.000	1.000	1.000
0.5	1.000	1.000	1.000	1.000	1.000

	$MAF_1 = 0.4, MAF_2 = 0.5$				
$P(D)$ / MAF_3	0.1	0.2	0.3	0.4	0.5
0.5	1.000	1.000	1.000	1.000	1.000

	$MAF_1 = 0.5, MAF_2 = 0.5$				
$P(D)$ / MAF_3	0.1	0.2	0.3	0.4	0.5
0.5	1.000	1.000	1.000	1.000	1.000

Table S12: The detailed table of successful generation frequencies for 3-order eNME model with $h^2 = 0.05$.

	$h^2=0.05, MAF_1=0.1, MAF_2=0.1$				
MAF_3 \ $P(D)$	0.1	0.2	0.3	0.4	0.5
0.1	1.000	1.000	1.000	0.950	0.010
0.2	1.000	1.000	1.000	1.000	0.200
0.3	1.000	1.000	1.000	0.700	0.000
0.4	1.000	1.000	0.555	0.000	0.000
0.5	1.000	1.000	0.000	0.000	0.000

	$h^2=0.05, MAF_1=0.1, MAF_2=0.2$				
MAF_3 \ $P(D)$	0.1	0.2	0.3	0.4	0.5
0.2	1.000	1.000	1.000	0.940	0.000
0.3	1.000	1.000	0.980	0.000	0.000
0.4	1.000	1.000	0.000	0.000	0.000
0.5	1.000	1.000	1.000	0.000	0.000

	$h^2=0.05, MAF_1=0.1, MAF_2=0.3$				
MAF_3 \ $P(D)$	0.1	0.2	0.3	0.4	0.5
0.3	1.000	1.000	0.000	0.000	0.000
0.4	1.000	0.500	0.000	0.000	0.000
0.5	1.000	0.000	0.000	0.000	0.000

	$h^2=0.05, MAF_1=0.1, MAF_2=0.4$				
MAF_3 \ $P(D)$	0.1	0.2	0.3	0.4	0.5
0.4	1.000	0.000	0.000	0.000	0.000
0.5	0.430	0.000	0.000	0.000	0.000

	$h^2=0.05, MAF_1=0.1, MAF_2=0.5$				
MAF_3 \ $P(D)$	0.1	0.2	0.3	0.4	0.5
0.5	1.000	0.000	0.000	0.000	0.000

	$h^2=0.05, MAF_1=0.2, MAF_2=0.2$				
MAF_3 \ $P(D)$	0.1	0.2	0.3	0.4	0.5
0.2	1.000	1.000	1.000	0.000	0.000
0.3	1.000	1.000	0.965	0.000	0.000
0.4	1.000	1.000	0.000	0.000	0.000
0.5	1.000	0.360	0.000	0.000	0.000

	$h^2=0.05, MAF_1=0.2, MAF_2=0.3$				
MAF_3 \ $P(D)$	0.1	0.2	0.3	0.4	0.5
0.3	1.000	1.000	0.115	0.000	0.000
0.4	1.000	0.905	0.000	0.000	0.000
0.5	1.000	0.000	0.000	0.000	0.000

	$h^2=0.05, MAF_1=0.2, MAF_2=0.4$				
MAF_3 \ $P(D)$	0.1	0.2	0.3	0.4	0.5
0.4	1.000	0.010	0.000	0.000	0.000
0.5	0.995	0.000	0.000	0.000	0.000

	$h^2=0.05, MAF_1=0.2, MAF_2=0.5$				
MAF_3 \ $P(D)$	0.1	0.2	0.3	0.4	0.5
0.5	0.017	0.000	0.000	0.000	0.000

	$h^2=0.05, MAF_1=0.3, MAF_2=0.3$				
MAF_3 \ $P(D)$	0.1	0.2	0.3	0.4	0.5
0.3	1.000	1.000	0.775	0.875	0.000
0.4	1.000	1.000	0.000	0.000	0.000
0.5	1.000	0.225	0.000	0.000	0.000

	$h^2=0.05, MAF_1=0.3, MAF_2=0.4$				
MAF_3 \ $P(D)$	0.1	0.2	0.3	0.4	0.5
0.4	0.000	0.000	0.000	0.000	0.000
0.5	1.000	0.965	0.000	0.000	0.000

	$h^2=0.05, MAF_1=0.3, MAF_2=0.5$				
MAF_3 \ $P(D)$	0.1	0.2	0.3	0.4	0.5
0.5	1.000	0.010	0.000	0.000	0.000

	$h^2=0.05, MAF_1=0.4, MAF_2=0.4$				
MAF_3 \ $P(D)$	0.1	0.2	0.3	0.4	0.5
0.4	1.000	1.000	0.000	0.965	0.000
0.5	1.000	0.990	0.000	0.003	0.000

	$h^2=0.05, MAF_1=0.4, MAF_2=0.5$				
MAF_3 \ $P(D)$	0.1	0.2	0.3	0.4	0.5
0.5	1.000	1.000	0.955	0.955	0.710

	$h^2=0.05, MAF_1=0.5, MAF_2=0.5$				
MAF_3 \ $P(D)$	0.1	0.2	0.3	0.4	0.5
0.5	1.000	1.000	0.730	1.000	0.790

Table S13: The detailed table of successful generation frequencies for 3-order eNME model with $h^2 = 0.1$.

	h^2=0.1, $MAF_1 = 0.1$, $MAF_2 = 0.1$				
$P(D)$ / MAF_3	0.1	0.2	0.3	0.4	0.5
0.1	1.000	1.000	1.000	0.000	0.001
0.2	1.000	1.000	1.000	0.005	0.200
0.3	1.000	1.000	1.000	0.710	0.000
0.4	1.000	1.000	0.555	0.000	0.000
0.5	1.000	0.997	0.000	0.000	0.000

	h^2=0.1, $MAF_1 = 0.1$, $MAF_2 = 0.2$				
$P(D)$ / MAF_3	0.1	0.2	0.3	0.4	0.5
0.2	1.000	1.000	1.000	0.940	0.000
0.3	1.000	1.000	0.985	0.000	0.000
0.4	1.000	1.000	0.000	0.000	0.000
0.5	1.000	1.000	1.000	0.000	0.000

	h^2=0.1, $MAF_1 = 0.1$, $MAF_2 = 0.3$				
$P(D)$ / MAF_3	0.1	0.2	0.3	0.4	0.5
0.3	1.000	1.000	0.000	0.000	0.000
0.4	1.000	0.040	0.000	0.000	0.000
0.5	1.000	0.000	0.000	0.000	0.000

	h^2=0.1, $MAF_1 = 0.1$, $MAF_2 = 0.4$				
$P(D)$ / MAF_3	0.1	0.2	0.3	0.4	0.5
0.4	1.000	0.000	0.000	0.000	0.000
0.5	0.460	0.000	0.000	0.000	0.000

	h^2=0.1, $MAF_1 = 0.1$, $MAF_2 = 0.5$				
$P(D)$ / MAF_3	0.1	0.2	0.3	0.4	0.5
0.5	0.001	0.000	0.000	0.000	0.000

	h^2=0.1, $MAF_1 = 0.2$, $MAF_2 = 0.2$				
$P(D)$ / MAF_3	0.1	0.2	0.3	0.4	0.5
0.2	1.000	1.000	1.000	0.995	0.000
0.3	1.000	1.000	0.990	0.000	0.000
0.4	1.000	1.000	0.000	0.000	0.000
0.5	1.000	0.250	0.000	0.000	0.000

	h^2=0.1, $MAF_1 = 0.2$, $MAF_2 = 0.3$				
$P(D)$ / MAF_3	0.1	0.2	0.3	0.4	0.5
0.3	1.000	1.000	0.170	0.000	0.000
0.4	1.000	0.940	0.000	0.000	0.000
0.5	1.000	0.001	0.000	0.000	0.000

	h^2=0.1, $MAF_1 = 0.2$, $MAF_2 = 0.4$				
$P(D)$ / MAF_3	0.1	0.2	0.3	0.4	0.5
0.4	1.000	0.000	0.000	0.000	0.000
0.5	0.995	0.000	0.000	0.000	0.000

	$MAF_1 = 0.2$, $MAF_2 = 0.5$				
$P(D)$ / MAF_3	0.1	0.2	0.3	0.4	0.5
0.5	0.290	0.000	0.000	0.000	0.000

	h^2=0.1, $MAF_1 = 0.3$, $MAF_2 = 0.3$				
$P(D)$ / MAF_3	0.1	0.2	0.3	0.4	0.5
0.3	1.000	1.000	0.830	0.130	0.000
0.4	1.000	1.000	0.000	0.000	0.000
0.5	1.000	0.300	0.000	0.000	0.000

	h^2=0.1, $MAF_1 = 0.3$, $MAF_2 = 0.4$				
$P(D)$ / MAF_3	0.1	0.2	0.3	0.4	0.5
0.4	0.000	0.000	0.000	0.000	0.000
0.5	1.000	1.000	0.000	0.000	0.000

	h^2=0.1, $MAF_1 = 0.3$, $MAF_2 = 0.5$				
$P(D)$ / MAF_3	0.1	0.2	0.3	0.4	0.5
0.5	1.000	0.100	0.000	0.000	0.000

	h^2=0.1, $MAF_1 = 0.4$, $MAF_2 = 0.4$				
$P(D)$ / MAF_3	0.1	0.2	0.3	0.4	0.5
0.4	1.000	0.995	0.000	0.050	0.000
0.5	1.000	1.000	0.000	0.000	0.000

	h^2=0.1, $MAF_1 = 0.4$, $MAF_2 = 0.5$				
$P(D)$ / MAF_3	0.1	0.2	0.3	0.4	0.5
0.5	1.000	1.000	0.935	0.935	0.425

	h^2=0.1, $MAF_1 = 0.5$, $MAF_2 = 0.5$				
$P(D)$ / MAF_3	0.1	0.2	0.3	0.4	0.5
0.5	1.000	1.000	0.665	0.915	0.000

Table S14: The detailed table of successful generation frequencies for 3-order eNME model with $h^2 = 0.15$.

	h^2=0.15, $MAF_1 = 0.1, MAF_2 = 0.1$				
MAF_3 \ $P(D)$	0.1	0.2	0.3	0.4	0.5
0.1	1.000	1.000	1.000	0.000	0.000
0.2	1.000	1.000	1.000	0.000	0.000
0.3	1.000	1.000	1.000	0.000	0.000
0.4	1.000	1.000	0.555	0.000	0.000
0.5	1.000	0.970	0.000	0.000	0.000

	h^2=0.15, $MAF_1 = 0.1, MAF_2 = 0.2$				
MAF_3 \ $P(D)$	0.1	0.2	0.3	0.4	0.5
0.2	1.000	1.000	1.000	0.800	0.000
0.3	1.000	1.000	0.985	0.000	0.000
0.4	1.000	1.000	0.000	0.000	0.000
0.5	1.000	0.020	0.000	0.000	0.000

	h^2=0.15, $MAF_1 = 0.1, MAF_2 = 0.3$				
MAF_3 \ $P(D)$	0.1	0.2	0.3	0.4	0.5
0.3	1.000	1.000	0.010	0.000	0.000
0.4	1.000	0.050	0.000	0.000	0.000
0.5	1.000	0.000	0.000	0.000	0.000

	h^2=0.15, $MAF_1 = 0.1, MAF_2 = 0.4$				
MAF_3 \ $P(D)$	0.1	0.2	0.3	0.4	0.5
0.4	1.000	0.000	0.000	0.000	0.000
0.5	0.440	0.000	0.000	0.000	0.000

	h^2=0.15, $MAF_1 = 0.1, MAF_2 = 0.5$				
MAF_3 \ $P(D)$	0.1	0.2	0.3	0.4	0.5
0.5	0.080	0.000	0.000	0.000	0.000

	h^2=0.15, $MAF_1 = 0.2, MAF_2 = 0.2$				
MAF_3 \ $P(D)$	0.1	0.2	0.3	0.4	0.5
0.2	1.000	1.000	1.000	0.850	0.000
0.3	1.000	1.000	0.990	0.000	0.000
0.4	1.000	1.000	0.000	0.000	0.000
0.5	1.000	0.250	0.000	0.000	0.000

	h^2=0.15, $MAF_1 = 0.2, MAF_2 = 0.3$				
MAF_3 \ $P(D)$	0.1	0.2	0.3	0.4	0.5
0.3	1.000	1.000	0.170	0.850	0.000
0.4	1.000	0.940	0.000	0.000	0.000
0.5	1.000	0.005	0.000	0.000	0.000

	h^2=0.15, $MAF_1 = 0.2, MAF_2 = 0.4$				
MAF_3 \ $P(D)$	0.1	0.2	0.3	0.4	0.5
0.4	1.000	0.000	0.000	0.000	0.000
0.5	0.995	0.000	0.000	0.000	0.000

	h^2=0.15, $MAF_1 = 0.2, MAF_2 = 0.5$				
MAF_3 \ $P(D)$	0.1	0.2	0.3	0.4	0.5
0.5	0.13	0.000	0.000	0.000	0.000

	h^2=0.15, $MAF_1 = 0.3, MAF_2 = 0.3$				
MAF_3 \ $P(D)$	0.1	0.2	0.3	0.4	0.5
0.3	1.000	1.000	0.525	0.000	0.000
0.4	1.000	1.000	0.000	0.000	0.000
0.5	1.000	0.250	0.000	0.000	0.000

	h^2=0.15, $MAF_1 = 0.3, MAF_2 = 0.4$				
MAF_3 \ $P(D)$	0.1	0.2	0.3	0.4	0.5
0.4	0.000	0.000	0.000	0.000	0.000
0.5	1.000	0.980	0.000	0.000	0.000

	h^2=0.15, $MAF_1 = 0.3, MAF_2 = 0.5$				
MAF_3 \ $P(D)$	0.1	0.2	0.3	0.4	0.5
0.5	1.000	0.001	0.000	0.000	0.000

	h^2=0.15, $MAF_1 = 0.4, MAF_2 = 0.4$				
MAF_3 \ $P(D)$	0.1	0.2	0.3	0.4	0.5
0.4	1.000	1.000	0.000	0.000	0.000
0.5	1.000	1.000	0.000	0.000	0.000

	h^2=0.15, $MAF_1 = 0.4, MAF_2 = 0.5$				
MAF_3 \ $P(D)$	0.1	0.2	0.3	0.4	0.5
0.5	1.000	1.000	0.930	0.750	0.050

	h^2=0.15, $MAF_1 = 0.5, MAF_2 = 0.5$				
MAF_3 \ $P(D)$	0.1	0.2	0.3	0.4	0.5
0.5	1.000	1.000	0.770	0.005	0.000

Table S15: The detailed table of successful generation frequencies for 3-order eNME model with $h^2 = 0.2$.

	$h^2=0.2, MAF_1=0.1, MAF_2=0.1$				
$P(D)$ / MAF_3	0.1	0.2	0.3	0.4	0.5
0.1	1.000	1.000	1.000	0.000	0.000
0.2	1.000	1.000	1.000	0.000	0.000
0.3	1.000	1.000	1.000	0.000	0.000
0.4	1.000	1.000	0.500	0.000	0.000
0.5	1.000	0.985	0.000	0.000	0.000

	$h^2=0.2, MAF_1=0.1, MAF_2=0.2$				
$P(D)$ / MAF_3	0.1	0.2	0.3	0.4	0.5
0.2	1.000	1.000	1.000	0.000	0.000
0.3	1.000	1.000	0.980	0.000	0.000
0.4	1.000	1.000	0.000	0.000	0.000
0.5	1.000	0.010	1.000	0.000	0.000

	$h^2=0.2, MAF_1=0.1, MAF_2=0.3$				
$P(D)$ / MAF_3	0.1	0.2	0.3	0.4	0.5
0.3	1.000	1.000	0.050	0.000	0.000
0.4	1.000	0.040	0.000	0.000	0.000
0.5	1.000	0.000	0.000	0.000	0.000

	$h^2=0.2, MAF_1=0.1, MAF_2=0.4$				
$P(D)$ / MAF_3	0.1	0.2	0.3	0.4	0.5
0.4	1.000	0.000	0.000	0.000	0.000
0.5	0.440	0.000	0.000	0.000	0.000

	$h^2=0.2, MAF_1=0.1, MAF_2=0.5$				
$P(D)$ / MAF_3	0.1	0.2	0.3	0.4	0.5
0.5	0.020	0.000	0.000	0.000	0.000

	$h^2=0.2, MAF_1=0.2, MAF_2=0.2$				
$P(D)$ / MAF_3	0.1	0.2	0.3	0.4	0.5
0.2	1.000	1.000	1.000	0.000	0.000
0.3	1.000	1.000	0.995	0.000	0.000
0.4	1.000	1.000	0.000	0.000	0.000
0.5	1.000	0.210	0.000	0.000	0.000

	$h^2=0.2, MAF_1=0.2, MAF_2=0.3$				
$P(D)$ / MAF_3	0.1	0.2	0.3	0.4	0.5
0.3	1.000	1.000	0.05	0.000	0.000
0.4	1.000	0.905	0.000	0.000	0.000
0.5	1.000	0.000	0.000	0.000	0.000

	$h^2=0.2, MAF_1=0.2, MAF_2=0.4$				
$P(D)$ / MAF_3	0.1	0.2	0.3	0.4	0.5
0.4	1.000	0.000	0.000	0.000	0.000
0.5	1.000	0.000	0.000	0.000	0.000

	$h^2=0.2, MAF_1=0.2, MAF_2=0.5$				
$P(D)$ / MAF_3	0.1	0.2	0.3	0.4	0.5
0.5	0.110	0.000	0.000	0.000	0.000

	$h^2=0.2, MAF_1=0.3, MAF_2=0.3$				
$P(D)$ / MAF_3	0.1	0.2	0.3	0.4	0.5
0.3	1.000	1.000	0.750	0.000	0.000
0.4	1.000	1.000	0.000	0.000	0.000
0.5	1.000	0.240	0.000	0.000	0.000

	$h^2=0.2, MAF_1=0.3, MAF_2=0.4$				
$P(D)$ / MAF_3	0.1	0.2	0.3	0.4	0.5
0.4	0.000	0.000	0.000	0.000	0.000
0.5	1.000	0.980	0.000	0.000	0.000

	$h^2=0.2, MAF_1=0.3, MAF_2=0.5$				
$P(D)$ / MAF_3	0.1	0.2	0.3	0.4	0.5
0.5	1.000	0.001	0.000	0.000	0.000

	$h^2=0.2, MAF_1=0.4, MAF_2=0.4$				
$P(D)$ / MAF_3	0.1	0.2	0.3	0.4	0.5
0.4	1.000	1.000	0.000	0.000	0.000
0.5	1.000	1.000	0.000	0.000	0.000

	$h^2=0.2, MAF_1=0.4, MAF_2=0.5$				
$P(D)$ / MAF_3	0.1	0.2	0.3	0.4	0.5
0.5	1.000	1.000	0.930	0.750	0.050

	$h^2=0.2, MAF_1=0.5, MAF_2=0.5$				
$P(D)$ / MAF_3	0.1	0.2	0.3	0.4	0.5
0.5	1.000	1.000	0.770	0.005	0.000

Table S16: The detailed table of successful generation frequencies for 3-order eNME model with $h^2 = 0.3$.

	h^2=0.3, $MAF_1 = 0.1, MAF_2 = 0.1$				
MAF_3 \ $P(D)$	0.1	0.2	0.3	0.4	0.5
0.1	1.000	1.000	0.000	0.000	0.000
0.2	1.000	1.000	0.000	0.000	0.000
0.3	1.000	1.000	0.000	0.000	0.000
0.4	1.000	1.000	0.000	0.000	0.000
0.5	1.000	0.985	0.000	0.000	0.000
	h^2=0.3, $MAF_1 = 0.1, MAF_2 = 0.2$				
MAF_3 \ $P(D)$	0.1	0.2	0.3	0.4	0.5
0.2	1.000	1.000	0.000	0.000	0.000
0.3	1.000	1.000	0.000	0.000	0.000
0.4	1.000	1.000	0.000	0.000	0.000
0.5	1.000	0.020	0.000	0.000	0.000
	h^2=0.3, $MAF_1 = 0.1, MAF_2 = 0.3$				
MAF_3 \ $P(D)$	0.1	0.2	0.3	0.4	0.5
0.3	1.000	1.000	0.000	0.000	0.000
0.4	1.000	0.035	0.000	0.000	0.000
0.5	1.000	0.000	0.000	0.000	0.000
	h^2=0.3, $MAF_1 = 0.1, MAF_2 = 0.4$				
MAF_3 \ $P(D)$	0.1	0.2	0.3	0.4	0.5
0.4	0.000	0.000	0.000	0.000	0.000
0.5	0.400	0.000	0.000	0.000	0.000
	h^2=0.3, $MAF_1 = 0.1, MAF_2 = 0.5$				
MAF_3 \ $P(D)$	0.1	0.2	0.3	0.4	0.5
0.5	0.010	0.000	0.000	0.000	0.000
	h^2=0.3, $MAF_1 = 0.2, MAF_2 = 0.2$				
MAF_3 \ $P(D)$	0.1	0.2	0.3	0.4	0.5
0.2	1.000	1.000	0.000	0.000	0.000
0.3	1.000	1.000	0.000	0.000	0.000
0.4	1.000	1.000	0.000	0.000	0.000
0.5	1.000	0.250	0.000	0.000	0.000
	h^2=0.3, $MAF_1 = 0.2, MAF_2 = 0.3$				
MAF_3 \ $P(D)$	0.1	0.2	0.3	0.4	0.5
0.3	1.000	1.000	0.004	0.000	0.000
0.4	1.000	0.905	0.000	0.000	0.000
0.5	1.000	0.000	0.000	0.000	0.000
	h^2=0.3, $MAF_1 = 0.2, MAF_2 = 0.4$				
MAF_3 \ $P(D)$	0.1	0.2	0.3	0.4	0.5
0.4	1.000	0.000	0.000	0.000	0.000
0.5	0.985	0.000	0.000	0.000	0.000
	h^2 = 0.3, $MAF_1 = 0.2, MAF_2 = 0.5$				
MAF_3 \ $P(D)$	0.1	0.2	0.3	0.4	0.5
0.5	0.130	0.000	0.000	0.000	0.000
	h^2=0.3, $MAF_1 = 0.3, MAF_2 = 0.3$				
MAF_3 \ $P(D)$	0.1	0.2	0.3	0.4	0.5
0.3	1.000	1.000	0.770	0.000	0.000
0.4	1.000	1.000	0.000	0.000	0.000
0.5	1.000	0.250	0.000	0.000	0.000
	h^2=0.3, $MAF_1 = 0.3, MAF_2 = 0.4$				
MAF_3 \ $P(D)$	0.1	0.2	0.3	0.4	0.5
0.4	0.000	1.000	0.000	0.000	0.000
0.5	1.000	0.980	0.000	0.000	0.000
	h^2=0.3, $MAF_1 = 0.3, MAF_2 = 0.5$				
MAF_3 \ $P(D)$	0.1	0.2	0.3	0.4	0.5
0.5	1.000	0.001	0.000	0.000	0.000
	h^2=0.3, $MAF_1 = 0.4, MAF_2 = 0.4$				
MAF_3 \ $P(D)$	0.1	0.2	0.3	0.4	0.5
0.4	1.000	1.000	0.000	0.000	0.000
0.5	1.000	1.000	0.000	0.000	0.000
	h^2=0.3, $MAF_1 = 0.4, MAF_2 = 0.5$				
MAF_3 \ $P(D)$	0.1	0.2	0.3	0.4	0.5
0.5	1.000	1.000	0.900	0.000	0.000
	h^2=0.3, $MAF_1 = 0.5, MAF_2 = 0.5$				
MAF_3 \ $P(D)$	0.1	0.2	0.3	0.4	0.5
0.5	1.000	1.000	0.720	0.000	0.000

Table S17: The detailed table of successful generation frequencies for 4-order eNME model with only prevalence specified.

	$MAF_1 = 0.1, MAF_2 = 0.1, MAF_3 = 0.1$				
MAF_4 \ $P(D)$	0.1	0.2	0.3	0.4	0.5
0.1	1.000	1.000	1.000	1.000	1.000
0.2	1.000	1.000	0.995	1.000	1.000
0.3	1.000	1.000	1.000	1.000	1.000
0.4	1.000	1.000	1.000	1.000	0.995
0.5	1.000	1.000	1.000	1.000	0.995

	$MAF_1 = 0.1, MAF_2 = 0.1, MAF_3 = 0.2$				
MAF_4 \ $P(D)$	0.1	0.2	0.3	0.4	0.5
0.2	1.000	1.000	1.000	1.000	0.000
0.3	1.000	1.000	1.000	1.000	0.000
0.4	1.000	1.000	1.000	1.000	0.000
0.5	1.000	1.000	1.000	0.985	0.000

	$MAF_1 = 0.1, MAF_2 = 0.1, MAF_3 = 0.3$				
MAF_4 \ $P(D)$	0.1	0.2	0.3	0.4	0.5
0.3	1.000	1.000	1.000	0.000	0.000
0.4	1.000	1.000	0.995	0.000	0.000
0.5	1.000	1.000	0.920	0.000	0.000

	$MAF_1 = 0.1, MAF_2 = 0.1, MAF_3 = 0.4$				
MAF_4 \ $P(D)$	0.1	0.2	0.3	0.4	0.5
0.4	1.000	1.000	0.000	0.000	0.000
0.5	1.000	0.990	0.000	0.000	0.000

	$MAF_1 = 0.1, MAF_2 = 0.1, MAF_3 = 0.5$				
MAF_4 \ $P(D)$	0.1	0.2	0.3	0.4	0.5
0.5	0.001	0.000	0.000	0.000	0.000

	$MAF_1 = 0.1, MAF_2 = 0.2, MAF_3 = 0.2$				
MAF_4 \ $P(D)$	0.1	0.2	0.3	0.4	0.5
0.2	1.000	1.000	1.000	0.975	0.000
0.3	1.000	1.000	1.000	0.950	0.025
0.4	1.000	1.000	0.995	0.990	0.005
0.5	1.000	1.000	1.000	0.995	0.000

	$MAF_1 = 0.1, MAF_2 = 0.2, MAF_3 = 0.3$				
MAF_4 \ $P(D)$	0.1	0.2	0.3	0.4	0.5
0.3	1.000	1.000	1.000	0.000	0.000
0.4	1.000	1.000	1.000	0.000	0.000
0.5	1.000	1.000	0.945	0.000	0.000

	$MAF_1 = 0.1, MAF_2 = 0.2, MAF_3 = 0.4$				
MAF_4 \ $P(D)$	0.1	0.2	0.3	0.4	0.5
0.4	1.000	1.000	0.000	0.000	0.000
0.5	1.000	1.000	0.000	0.000	0.000

	$MAF_1 = 0.1, MAF_2 = 0.2, MAF_3 = 0.5$				
MAF_4 \ $P(D)$	0.1	0.2	0.3	0.4	0.5
0.5	1.000	0.000	0.000	0.000	0.000

	$MAF_1 = 0.1, MAF_2 = 0.3, MAF_3 = 0.3$				
MAF_4 \ $P(D)$	0.1	0.2	0.3	0.4	0.5
0.3	1.000	0.400	0.005	0.000	0.000
0.4	1.000	0.310	0.000	0.000	0.000
0.5	1.000	0.200	0.000	0.000	0.000

	$MAF_1 = 0.1, MAF_2 = 0.3, MAF_3 = 0.4$				
MAF_4 \ $P(D)$	0.1	0.2	0.3	0.4	0.5
0.4	1.000	0.000	0.000	0.000	0.000
0.5	1.000	0.000	0.000	0.000	0.000

	$MAF_1 = 0.1, MAF_2 = 0.3, MAF_3 = 0.5$				
MAF_4 \ $P(D)$	0.1	0.2	0.3	0.4	0.5
0.5	0.150	0.000	0.000	0.000	0.000

	$MAF_1 = 0.1, MAF_2 = 0.4, MAF_3 = 0.4$				
MAF_4 \ $P(D)$	0.1	0.2	0.3	0.4	0.5
0.4	0.075	0.000	0.000	0.000	0.000
0.5	0.150	0.000	0.000	0.000	0.000

	$MAF_1 = 0.1, MAF_2 = 0.4, MAF_3 = 0.5$				
MAF_4 \ $P(D)$	0.1	0.2	0.3	0.4	0.5
0.5	0.000	0.000	0.000	0.000	0.000

	$MAF_1 = 0.1, MAF_2 = 0.5, MAF_3 = 0.5$				
MAF_4 \ $P(D)$	0.1	0.2	0.3	0.4	0.5
0.5	0.000	0.000	0.000	0.000	0.000

	$MAF_1 = 0.2, MAF_2 = 0.2, MAF_3 = 0.2$				

$P(D)$ / MAF_4	0.1	0.2	0.3	0.4	0.5
0.2	1.000	1.000	1.000	1.000	0.000
0.3	1.000	1.000	1.000	0.985	0.005
0.4	1.000	1.000	1.000	0.990	0.000
0.5	1.000	1.000	1.000	0.975	0.000
	$MAF_1 = 0.2, MAF_2 = 0.2, MAF_3 = 0.3$				
$P(D)$ / MAF_4	0.1	0.2	0.3	0.4	0.5
0.3	1.000	1.000	1.000	0.000	0.000
0.4	1.000	1.000	0.985	0.000	0.000
0.5	1.000	1.000	0.860	0.000	0.000
	$MAF_1 = 0.2, MAF_2 = 0.2, MAF_3 = 0.4$				
$P(D)$ / MAF_4	0.1	0.2	0.3	0.4	0.5
0.4	1.000	0.975	0.000	0.000	0.000
0.5	1.000	0.925	0.000	0.000	0.000
	$MAF_1 = 0.2, MAF_2 = 0.2, MAF_3 = 0.5$				
$P(D)$ / MAF_4	0.1	0.2	0.3	0.4	0.5
0.5	1.000	0.005	0.000	0.000	0.000
	$MAF_1 = 0.2, MAF_2 = 0.3, MAF_3 = 0.3$				
$P(D)$ / MAF_4	0.1	0.2	0.3	0.4	0.5
0.3	1.000	0.725	0.005	0.000	0.000
0.4	1.000	0.700	0.000	0.000	0.000
0.5	1.000	0.815	0.000	0.000	0.000
	$MAF_1 = 0.2, MAF_2 = 0.3, MAF_3 = 0.4$				
$P(D)$ / MAF_4	0.1	0.2	0.3	0.4	0.5
0.4	1.000	0.000	0.000	0.000	0.000
0.5	1.000	0.005	0.000	0.000	0.000
	$MAF_1 = 0.2, MAF_2 = 0.3, MAF_3 = 0.5$				
$P(D)$ / MAF_4	0.1	0.2	0.3	0.4	0.5
0.5	0.150	0.000	0.000	0.000	0.000
	$MAF_1 = 0.2, MAF_2 = 0.4, MAF_3 = 0.4$				
$P(D)$ / MAF_4	0.1	0.2	0.3	0.4	0.5
0.4	0.375	0.000	0.000	0.000	0.000
0.5	0.455	0.000	0.000	0.000	0.000
	$MAF_1 = 0.2, MAF_2 = 0.4, MAF_3 = 0.5$				

$P(D)$ / MAF_4	0.1	0.2	0.3	0.4	0.5
0.5	0.000	0.000	0.000	0.000	0.000
	$MAF_1 = 0.2, MAF_2 = 0.5, MAF_3 = 0.5$				
$P(D)$ / MAF_4	0.1	0.2	0.3	0.4	0.5
0.5	0.000	0.000	0.000	0.000	0.000
	$MAF_1 = 0.3, MAF_2 = 0.3, MAF_3 = 0.3$				
$P(D)$ / MAF_4	0.1	0.2	0.3	0.4	0.5
0.3	1.000	0.760	0.001	0.000	0.000
0.4	1.000	0.710	0.001	0.000	0.000
0.5	1.000	0.685	0.000	0.000	0.000
	$MAF_1 = 0.3, MAF_2 = 0.3, MAF_3 = 0.4$				
$P(D)$ / MAF_4	0.1	0.2	0.3	0.4	0.5
0.4	1.000	0.000	0.000	0.000	0.000
0.5	1.000	0.015	0.000	0.000	0.000
	$MAF_1 = 0.3MAF_2 = 0.3, MAF_3 = 0.5$				
$P(D)$ / MAF_4	0.1	0.2	0.3	0.4	0.5
0.5	0.200	0.000	0.000	0.000	0.000
	$MAF_1 = 0.3, MAF_2 = 0.4, MAF_3 = 0.4$				
$P(D)$ / MAF_4	0.1	0.2	0.3	0.4	0.5
0.4	0.430	0.000	0.000	0.000	0.000
0.5	0.445	0.000	0.000	0.000	0.000
	$MAF_1 = 0.3, MAF_2 = 0.4, MAF_3 = 0.5$				
$P(D)$ / MAF_4	0.1	0.2	0.3	0.4	0.5
0.5	0.000	0.000	0.000	0.000	0.000
	$MAF_1 = 0.3, MAF_2 = 0.5, MAF_3 = 0.5$				
$P(D)$ / MAF_4	0.1	0.2	0.3	0.4	0.5
0.5	0.000	0.000	0.000	0.000	0.000
	$MAF_1 = 0.4, MAF_2 = 0.4, MAF_3 = 0.4$				
$P(D)$ / MAF_4	0.1	0.2	0.3	0.4	0.5
0.4	0.000	0.000	0.000	0.000	0.000
0.5	0.000	0.000	0.000	0.000	0.000
	$MAF_1 = 0.4, MAF_2 = 0.4, MAF_3 = 0.5$				

$P(D)$ / MAF_4	0.1	0.2	0.3	0.4	0.5
0.5	0.000	0.000	0.000	0.000	0.000
	$MAF_1 = 0.4, MAF_2 = 0.5, MAF_3 = 0.5$				
$P(D)$ / MAF_4	0.1	0.2	0.3	0.4	0.5
0.5	0.000	0.000	0.000	0.000	0.000
	$MAF_1 = 0.5, MAF_2 = 0.5, MAF_3 = 0.5$				
$P(D)$ / MAF_4	0.1	0.2	0.3	0.4	0.5
0.5	0.000	0.000	0.000	0.000	0.000

Table S18: The detailed table of successful generation frequencies for 4-order eNME model with $h^2 = 0.05$.

	$h^2=0.05, MAF_1=0.1, MAF_2=0.1, MAF_3=0.1$				
MAF_4 \ $P(D)$	0.1	0.2	0.3	0.4	0.5
0.1	1.000	0.050	0.015	0.000	0.000
0.2	1.000	0.005	0.001	0.000	0.000
0.3	1.000	0.000	0.005	0.000	0.000
0.4	0.710	0.000	0.000	0.000	0.000
0.5	0.020	0.000	0.000	0.000	0.000
	$h^2=0.05, MAF_1=0.1, MAF_2=0.1, MAF_3=0.2$				
MAF_4 \ $P(D)$	0.1	0.2	0.3	0.4	0.5
0.2	1.000	0.001	0.000	0.000	0.000
0.3	1.000	0.000	0.000	0.000	0.000
0.4	0.480	0.000	0.000	0.000	0.000
0.5	0.010	0.000	0.000	0.000	0.000
	$h^2=0.05, MAF_1=0.1, MAF_2=0.1, MAF_3=0.3$				
MAF_4 \ $P(D)$	0.1	0.2	0.3	0.4	0.5
0.3	1.000	0.000	0.000	0.000	0.000
0.4	0.050	0.000	0.000	0.000	0.000
0.5	0.000	0.000	0.000	0.000	0.000
	$h^2=0.05, MAF_1=0.1, MAF_2=0.1, MAF_3=0.4$				
MAF_4 \ $P(D)$	0.1	0.2	0.3	0.4	0.5
0.4	0.000	0.000	0.000	0.000	0.000
0.5	0.000	0.000	0.000	0.000	0.000
	$h^2=0.05, MAF_1=0.1, MAF_2=0.1, MAF_3=0.5$				
MAF_4 \ $P(D)$	0.1	0.2	0.3	0.4	0.5
0.5	0.000	0.000	0.000	0.000	0.000
	$h^2=0.05, MAF_1=0.1, MAF_2=0.2, MAF_3=0.2$				
MAF_4 \ $P(D)$	0.1	0.2	0.3	0.4	0.5
0.2	1.000	0.015	0.000	0.000	0.000
0.3	1.000	0.000	0.000	0.000	0.000
0.4	1.000	0.000	0.000	0.000	0.000
0.5	0.925	0.000	0.000	0.000	0.000
	$h^2=0.05, MAF_1=0.1, MAF_2=0.2, MAF_3=0.3$				
MAF_4 \ $P(D)$	0.1	0.2	0.3	0.4	0.5
0.3	1.000	0.004	0.000	0.000	0.000
0.4	1.000	0.000	0.000	0.000	0.000
0.5	0.070	0.000	0.000	0.000	0.000
	$h^2=0.05, MAF_1=0.1, MAF_2=0.2, MAF_3=0.4$				
MAF_4 \ $P(D)$	0.1	0.2	0.3	0.4	0.5
0.4	0.100	0.000	0.000	0.000	0.000
0.5	0.000	0.000	0.000	0.000	0.000
	$h^2=0.05, MAF_1=0.1, MAF_2=0.2, MAF_3=0.5$				
MAF_4 \ $P(D)$	0.1	0.2	0.3	0.4	0.5
0.5	0.001	0.000	0.000	0.000	0.000
	$h^2=0.05, MAF_1=0.1, MAF_2=0.3, MAF_3=0.3$				
MAF_4 \ $P(D)$	0.1	0.2	0.3	0.4	0.5
0.3	0.410	0.000	0.000	0.000	0.000
0.4	0.440	0.000	0.000	0.000	0.000
0.5	0.000	0.000	0.000	0.000	0.000
	$h^2=0.05, MAF_1=0.1, MAF_2=0.3, MAF_3=0.4$				
MAF_4 \ $P(D)$	0.1	0.2	0.3	0.4	0.5
0.4	0.390	0.000	0.000	0.000	0.000
0.5	0.320	0.000	0.000	0.000	0.000
	$h^2=0.05, MAF_1=0.1, MAF_2=0.3, MAF_3=0.5$				
MAF_4 \ $P(D)$	0.1	0.2	0.3	0.4	0.5
0.5	0.001	0.000	0.000	0.000	0.000
	$h^2=0.05, MAF_1=0.1, MAF_2=0.4, MAF_3=0.4$				
MAF_4 \ $P(D)$	0.1	0.2	0.3	0.4	0.5
0.4	0.040	0.000	0.000	0.000	0.000
0.5	0.040	0.000	0.000	0.000	0.000
	$h^2=0.05, MAF_1=0.1, MAF_2=0.4, MAF_3=0.5$				
MAF_4 \ $P(D)$	0.1	0.2	0.3	0.4	0.5
0.5	0.000	0.000	0.000	0.000	0.000
	$h^2=0.05, MAF_1=0.1, MAF_2=0.5, MAF_3=0.5$				
MAF_4 \ $P(D)$	0.1	0.2	0.3	0.4	0.5
0.5	0.000	0.000	0.000	0.000	0.000
	$h^2=0.05, MAF_1=0.2, MAF_2=0.2, MAF_3=0.2$				
MAF_4 \ $P(D)$	0.1	0.2	0.3	0.4	0.5

0.2	1.000	0.001	0.000	0.000	0.000
0.3	1.000	0.035	0.000	0.000	0.000
0.4	0.315	0.001	0.000	0.000	0.000
0.5	0.000	0.002	0.000	0.000	0.000

	$h^2 = 0.05, MAF_1 = 0.2, MAF_2 = 0.2, MAF_3 = 0.3$				
$P(D)$ / MAF_4	0.1	0.2	0.3	0.4	0.5
0.3	1.000	0.000	0.000	0.000	0.000
0.4	0.005	0.001	0.000	0.000	0.000
0.5	0.000	0.000	0.000	0.000	0.000

	$h^2 = 0.05, MAF_1 = 0.1, MAF_2 = 0.2, MAF_3 = 0.4$				
$P(D)$ / MAF_4	0.1	0.2	0.3	0.4	0.5
0.4	0.001	0.000	0.000	0.000	0.000
0.5	0.000	0.000	0.000	0.000	0.000

	$h^2 = 0.05, MAF_1 = 0.2, MAF_2 = 0.5, MAF_3 = 0.5$				
$P(D)$ / MAF_4	0.1	0.2	0.3	0.4	0.5
0.5	0.025	0.000	0.000	0.000	0.000

	$h^2 = 0.05, MAF_1 = 0.2, MAF_2 = 0.3, MAF_3 = 0.3$				
$P(D)$ / MAF_4	0.1	0.2	0.3	0.4	0.5
0.3	0.985	0.000	0.000	0.000	0.000
0.4	0.980	0.000	0.000	0.000	0.000
0.5	0.985	0.000	0.000	0.000	0.000

	$h^2 = 0.05, MAF_1 = 0.2, MAF_2 = 0.3, MAF_3 = 0.4$				
$P(D)$ / MAF_4	0.1	0.2	0.3	0.4	0.5
0.4	0.765	0.000	0.000	0.000	0.000
0.5	0.220	0.000	0.000	0.000	0.000

	$h^2 = 0.05, MAF_1 = 0.2, MAF_2 = 0.3, MAF_3 = 0.5$				
$P(D)$ / MAF_4	0.1	0.2	0.3	0.4	0.5
0.5	0.000	0.000	0.000	0.000	0.000

	$h^2 = 0.05, MAF_1 = 0.2, MAF_2 = 0.4, MAF_3 = 0.4$				
$P(D)$ / MAF_4	0.1	0.2	0.3	0.4	0.5
0.4	0.003	0.000	0.000	0.000	0.000
0.5	0.000	0.000	0.000	0.000	0.000

	$h^2 = 0.05, MAF_1 = 0.2, MAF_2 = 0.4, MAF_3 = 0.5$				
$P(D)$ / MAF_4	0.1	0.2	0.3	0.4	0.5
0.5	0.000	0.000	0.000	0.000	0.000

	$h^2 = 0.05, MAF_1 = 0.2, MAF_2 = 0.5, MAF_3 = 0.5$				
$P(D)$ / MAF_4	0.1	0.2	0.3	0.4	0.5
0.5	0.000	0.000	0.000	0.000	0.000

	$h^2 = 0.05, MAF_1 = 0.3, MAF_2 = 0.3, MAF_3 = 0.3$				
$P(D)$ / MAF_4	0.1	0.2	0.3	0.4	0.5
0.3	0.090	0.000	0.000	0.000	0.000
0.4	0.825	0.000	0.000	0.000	0.000
0.5	0.915	0.000	0.000	0.000	0.000

	$h^2 = 0.05, MAF_1 = 0.3, MAF_2 = 0.3, MAF_3 = 0.4$				
$P(D)$ / MAF_4	0.1	0.2	0.3	0.4	0.5
0.4	0.570	0.000	0.000	0.000	0.000
0.5	0.510	0.000	0.000	0.000	0.000

	$h^2 = 0.05, MAF_1 = 0.3, MAF_2 = 0.3, MAF_3 = 0.5$				
$P(D)$ / MAF_4	0.1	0.2	0.3	0.4	0.5
0.5	0.000	0.000	0.000	0.000	0.000

	$h^2 = 0.05, MAF_1 = 0.3, MAF_2 = 0.4, MAF_3 = 0.4$				
$P(D)$ / MAF_4	0.1	0.2	0.3	0.4	0.5
0.4	0.670	0.000	0.000	0.000	0.000
0.5	0.030	0.000	0.000	0.000	0.000

	$h^2 = 0.05, MAF_1 = 0.3, MAF_2 = 0.4, MAF_3 = 0.5$				
$P(D)$ / MAF_4	0.1	0.2	0.3	0.4	0.5
0.5	0.000	0.000	0.000	0.000	0.000

	$h^2 = 0.05, MAF_1 = 0.3, MAF_2 = 0.5, MAF_3 = 0.5$				
$P(D)$ / MAF_4	0.1	0.2	0.3	0.4	0.5
0.5	0.000	0.000	0.000	0.000	0.000

	$h^2 = 0.05, MAF_1 = 0.4, MAF_2 = 0.4, MAF_3 = 0.4$				
$P(D)$ / MAF_4	0.1	0.2	0.3	0.4	0.5
0.4	0.670	0.000	0.000	0.000	0.000
0.5	0.030	0.000	0.000	0.000	0.000

	$h^2 = 0.05, MAF_1 = 0.4, MAF_2 = 0.4, MAF_3 = 0.5$				
$P(D)$ / MAF_4	0.1	0.2	0.3	0.4	0.5
0.5	0.050	0.000	0.000	0.000	0.000

	$h^2 = 0.05, MAF_1 = 0.4, MAF_2 = 0.5, MAF_3 = 0.5$				

MAF_4 \ $P(D)$	0.1	0.2	0.3	0.4	0.5
0.5	0.000	0.000	0.000	0.000	0.000
	$h^2 = 0.05, MAF_1 = 0.5, MAF_2 = 0.5 MAF_3 = 0.5$				
MAF_4 \ $P(D)$	0.1	0.2	0.3	0.4	0.5
0.5	0.005	0.000	0.000	0.000	0.000

Table S19: The detailed table of successful generation frequencies for 4-order eNME model with $h^2 = 0.1$.

	$h^2=0.1, MAF_1=0.1, MAF_2=0.1, MAF_3=0.1$				
MAF_4 \ $P(D)$	0.1	0.2	0.3	0.4	0.5
0.1	0.000	0.025	0.000	0.000	0.000
0.2	1.000	0.005	0.000	0.000	0.000
0.3	1.000	0.000	0.000	0.000	0.000
0.4	0.440	0.000	0.000	0.000	0.000
0.5	0.010	0.000	0.000	0.000	0.000

	$h^2=0.1, MAF_1=0.1, MAF_2=0.1, MAF_3=0.2$				
MAF_4 \ $P(D)$	0.1	0.2	0.3	0.4	0.5
0.2	1.000	0.015	0.000	0.000	0.000
0.3	1.000	0.000	0.000	0.000	0.000
0.4	0.425	0.000	0.000	0.000	0.000
0.5	0.015	0.000	0.000	0.000	0.000

	$h^2=0.1, MAF_1=0.1, MAF_2=0.1, MAF_3=0.3$				
MAF_4 \ $P(D)$	0.1	0.2	0.3	0.4	0.5
0.3	1.000	0.000	0.000	0.000	0.000
0.4	0.030	0.000	0.000	0.000	0.000
0.5	0.000	0.000	0.000	0.000	0.000

	$h^2=0.1, MAF_1=0.1, MAF_2=0.1, MAF_3=0.4$				
MAF_4 \ $P(D)$	0.1	0.2	0.3	0.4	0.5
0.4	0.001	0.000	0.000	0.000	0.000
0.5	0.000	0.000	0.000	0.000	0.000

	$h^2=0.1, MAF_1=0.1, MAF_2=0.1, MAF_3=0.5$				
MAF_4 \ $P(D)$	0.1	0.2	0.3	0.4	0.5
0.5	0.000	0.000	0.000	0.000	0.000

	$h^2=0.1, MAF_1=0.1, MAF_2=0.2, MAF_3=0.2$				
MAF_4 \ $P(D)$	0.1	0.2	0.3	0.4	0.5
0.2	1.000	0.420	0.000	0.000	0.000
0.3	1.000	0.270	0.000	0.000	0.000
0.4	1.000	0.000	0.000	0.000	0.000
0.5	0.900	0.000	0.000	0.000	0.000

	$h^2=0.1, MAF_1=0.1, MAF_2=0.2, MAF_3=0.3$				
MAF_4 \ $P(D)$	0.1	0.2	0.3	0.4	0.5
0.3	1.000	0.001	0.000	0.000	0.000
0.4	1.000	0.000	0.000	0.000	0.000
0.5	0.070	0.000	0.000	0.000	0.000

	$h^2=0.1, MAF_1=0.1, MAF_2=0.2, MAF_3=0.4$				
MAF_4 \ $P(D)$	0.1	0.2	0.3	0.4	0.5
0.4	0.000	0.000	0.000	0.000	0.000
0.5	0.000	0.000	0.000	0.000	0.000

	$h^2=0.1, MAF_1=0.1, MAF_2=0.2, MAF_3=0.5$				
MAF_4 \ $P(D)$	0.1	0.2	0.3	0.4	0.5
0.5	0.000	0.000	0.000	0.000	0.000

	$h^2=0.1, MAF_1=0.1, MAF_2=0.3, MAF_3=0.3$				
MAF_4 \ $P(D)$	0.1	0.2	0.3	0.4	0.5
0.3	0.785	0.000	0.000	0.000	0.000
0.4	0.275	0.000	0.000	0.000	0.000
0.5	0.000	0.000	0.000	0.000	0.000

	$h^2=0.1, MAF_1=0.1, MAF_2=0.3, MAF_3=0.4$				
MAF_4 \ $P(D)$	0.1	0.2	0.3	0.4	0.5
0.4	0.225	0.000	0.000	0.000	0.000
0.5	0.045	0.000	0.000	0.000	0.000

	$h^2=0.1, MAF_1=0.1, MAF_2=0.3, MAF_3=0.5$				
MAF_4 \ $P(D)$	0.1	0.2	0.3	0.4	0.5
0.5	0.001	0.000	0.000	0.000	0.000

	$h^2=0.1, MAF_1=0.1, MAF_2=0.4, MAF_3=0.4$				
MAF_4 \ $P(D)$	0.1	0.2	0.3	0.4	0.5
0.4	0.055	0.000	0.000	0.000	0.000
0.5	0.070	0.000	0.000	0.000	0.000

	$h^2=0.1, MAF_1=0.1, MAF_2=0.4, MAF_3=0.5$				
MAF_4 \ $P(D)$	0.1	0.2	0.3	0.4	0.5
0.5	0.001	0.000	0.000	0.000	0.000

	$h^2=0.1, MAF_1=0.1, MAF_2=0.5, MAF_3=0.5$				
MAF_4 \ $P(D)$	0.1	0.2	0.3	0.4	0.5
0.5	0.000	0.000	0.000	0.000	0.000

	$h^2=0.1, MAF_1=0.2, MAF_2=0.2, MAF_3=0.2$				
MAF_4 \ $P(D)$	0.1	0.2	0.3	0.4	0.5

0.2	1.000	0.000	0.000	0.000	0.000
0.3	1.000	0.000	0.000	0.000	0.000
0.4	0.250	0.000	0.000	0.000	0.000
0.5	0.000	0.000	0.000	0.000	0.000

	$h^2 = 0.1, MAF_1 = 0.2, MAF_2 = 0.2, MAF_3 = 0.3$				
$P(D)$ / MAF_4	0.1	0.2	0.3	0.4	0.5
0.3	1.000	0.012	0.000	0.000	0.000
0.4	0.025	0.007	0.000	0.000	0.000
0.5	0.000	0.012	0.000	0.000	0.000

	$h^2 = 0.1, MAF_1 = 0.1, MAF_2 = 0.2, MAF_3 = 0.4$				
$P(D)$ / MAF_4	0.1	0.2	0.3	0.4	0.5
0.4	0.290	0.000	0.000	0.000	0.000
0.5	0.035	0.000	0.000	0.000	0.000

	$h^2 = 0.1, MAF_1 = 0.2, MAF_2 = 0.5, MAF_3 = 0.5$				
$P(D)$ / MAF_4	0.1	0.2	0.3	0.4	0.5
0.5	0.004	0.000	0.000	0.000	0.000

	$h^2 = 0.1, MAF_1 = 0.2, MAF_2 = 0.3, MAF_3 = 0.3$				
$P(D)$ / MAF_4	0.1	0.2	0.3	0.4	0.5
0.3	1.000	0.020	0.000	0.000	0.000
0.4	0.950	0.005	0.000	0.000	0.000
0.5	0.960	0.005	0.000	0.000	0.000

	$h^2 = 0.1, MAF_1 = 0.2, MAF_2 = 0.3, MAF_3 = 0.4$				
$P(D)$ / MAF_4	0.1	0.2	0.3	0.4	0.5
0.4	0.990	0.000	0.000	0.000	0.000
0.5	0.400	0.000	0.000	0.000	0.000

	$h^2 = 0.1, MAF_1 = 0.2, MAF_2 = 0.3, MAF_3 = 0.5$				
$P(D)$ / MAF_4	0.1	0.2	0.3	0.4	0.5
0.5	0.000	0.000	0.000	0.000	0.000

	$h^2 = 0.1, MAF_1 = 0.2, MAF_2 = 0.4, MAF_3 = 0.4$				
$P(D)$ / MAF_4	0.1	0.2	0.3	0.4	0.5
0.4	0.000	0.000	0.000	0.000	0.000
0.5	0.000	0.000	0.000	0.000	0.000

	$h^2 = 0.1, MAF_1 = 0.2, MAF_2 = 0.4, MAF_3 = 0.5$				
$P(D)$ / MAF_4	0.1	0.2	0.3	0.4	0.5
0.5	0.000	0.000	0.000	0.000	0.000

	$h^2 = 0.1, MAF_1 = 0.2, MAF_2 = 0.5, MAF_3 = 0.5$				
$P(D)$ / MAF_4	0.1	0.2	0.3	0.4	0.5
0.5	0.000	0.000	0.000	0.000	0.000

	$h^2 = 0.1, MAF_1 = 0.3, MAF_2 = 0.3, MAF_3 = 0.3$				
$P(D)$ / MAF_4	0.1	0.2	0.3	0.4	0.5
0.3	1.000	0.000	0.000	0.000	0.000
0.4	0.970	0.000	0.000	0.000	0.000
0.5	0.980	0.000	0.000	0.000	0.000

	$h^2 = 0.1, MAF_1 = 0.3, MAF_2 = 0.3, MAF_3 = 0.4$				
$P(D)$ / MAF_4	0.1	0.2	0.3	0.4	0.5
0.4	0.925	0.000	0.000	0.000	0.000
0.5	0.920	0.000	0.000	0.000	0.000

	$h^2 = 0.1, MAF_1 = 0.3, MAF_2 = 0.3, MAF_3 = 0.5$				
$P(D)$ / MAF_4	0.1	0.2	0.3	0.4	0.5
0.5	0.040	0.000	0.000	0.000	0.000

	$h^2 = 0.1, MAF_1 = 0.3, MAF_2 = 0.4, MAF_3 = 0.4$				
$P(D)$ / MAF_4	0.1	0.2	0.3	0.4	0.5
0.4	0.000	0.000	0.000	0.000	0.000
0.5	0.002	0.000	0.000	0.000	0.000

	$h^2 = 0.1, MAF_1 = 0.3, MAF_2 = 0.4, MAF_3 = 0.5$				
$P(D)$ / MAF_4	0.1	0.2	0.3	0.4	0.5
0.5	0.000	0.000	0.000	0.000	0.000

	$h^2 = 0.1, MAF_1 = 0.3, MAF_2 = 0.5, MAF_3 = 0.5$				
$P(D)$ / MAF_4	0.1	0.2	0.3	0.4	0.5
0.5	0.000	0.000	0.000	0.000	0.000

	$h^2 = 0.1, MAF_1 = 0.4, MAF_2 = 0.4, MAF_3 = 0.4$				
$P(D)$ / MAF_4	0.1	0.2	0.3	0.4	0.5
0.4	0.510	0.000	0.000	0.000	0.000
0.5	0.025	0.000	0.000	0.000	0.000

	$h^2 = 0.1, MAF_1 = 0.4, MAF_2 = 0.4, MAF_3 = 0.5$				
$P(D)$ / MAF_4	0.1	0.2	0.3	0.4	0.5
0.5	0.000	0.000	0.000	0.000	0.000

	$h^2 = 0.1, MAF_1 = 0.4, MAF_2 = 0.5, MAF_3 = 0.5$				

$P(D)$ / MAF_4	0.1	0.2	0.3	0.4	0.5
0.5	0.000	0.000	0.000	0.000	0.000
	$h^2 = 0.1, MAF_1 = 0.5, MAF_2 = 0.5 MAF_3 = 0.5$				
$P(D)$ / MAF_4	0.1	0.2	0.3	0.4	0.5
0.5	0.000	0.000	0.000	0.000	0.000

Table S20: The detailed table of successful generation frequencies for 4-order eNME model with $h^2 = 0.15$.

	$h^2 = 0.15, MAF_1 = 0.1, MAF_2 = 0.1, MAF_3 = 0.1$				
$P(D)$ / MAF_4	0.1	0.2	0.3	0.4	0.5
0.1	1.000	0.035	0.000	0.000	0.000
0.2	1.000	0.000	0.000	0.000	0.000
0.3	1.000	0.000	0.000	0.000	0.000
0.4	0.017	0.000	0.000	0.000	0.000
0.5	0.005	0.000	0.000	0.000	0.000

	$h^2 = 0.15, MAF_1 = 0.1, MAF_2 = 0.1, MAF_3 = 0.2$				
$P(D)$ / MAF_4	0.1	0.2	0.3	0.4	0.5
0.2	1.000	0.025	0.000	0.000	0.000
0.3	1.000	0.000	0.000	0.000	0.000
0.4	0.335	0.000	0.000	0.000	0.000
0.5	0.001	0.000	0.000	0.000	0.000

	$h^2 = 0.15, MAF_1 = 0.1, MAF_2 = 0.1, MAF_3 = 0.3$				
$P(D)$ / MAF_4	0.1	0.2	0.3	0.4	0.5
0.3	1.000	0.000	0.000	0.000	0.000
0.4	0.025	0.000	0.000	0.000	0.000
0.5	0.000	0.000	0.000	0.000	0.000

	$h^2 = 0.15, MAF_1 = 0.1, MAF_2 = 0.1, MAF_3 = 0.4$				
$P(D)$ / MAF_4	0.1	0.2	0.3	0.4	0.5
0.4	0.010	0.000	0.000	0.000	0.000
0.5	0.000	0.000	0.000	0.000	0.000

	$h^2 = 0.15, MAF_1 = 0.1, MAF_2 = 0.1, MAF_3 = 0.5$				
$P(D)$ / MAF_4	0.1	0.2	0.3	0.4	0.5
0.5	0.000	0.000	0.000	0.000	0.000

	$h^2 = 0.15, MAF_1 = 0.1, MAF_2 = 0.2, MAF_3 = 0.2$				
$P(D)$ / MAF_4	0.1	0.2	0.3	0.4	0.5
0.2	1.000	0.965	0.000	0.000	0.000
0.3	1.000	0.725	0.000	0.000	0.000
0.4	1.000	0.000	0.000	0.000	0.000
0.5	0.920	0.000	0.000	0.000	0.000

	$h^2 = 0.15, MAF_1 = 0.1, MAF_2 = 0.2, MAF_3 = 0.3$				
$P(D)$ / MAF_4	0.1	0.2	0.3	0.4	0.5
0.3	1.000	0.020	0.000	0.000	0.000
0.4	1.000	0.000	0.000	0.000	0.000
0.5	0.070	0.000	0.000	0.000	0.000

	$h^2 = 0.15, MAF_1 = 0.1, MAF_2 = 0.2, MAF_3 = 0.4$				
$P(D)$ / MAF_4	0.1	0.2	0.3	0.4	0.5
0.4	0.070	0.000	0.000	0.000	0.000
0.5	0.000	0.000	0.000	0.000	0.000

	$h^2 = 0.15, MAF_1 = 0.1, MAF_2 = 0.2, MAF_3 = 0.5$				
$P(D)$ / MAF_4	0.1	0.2	0.3	0.4	0.5
0.5	0.000	0.000	0.000	0.000	0.000

	$h^2 = 0.15, MAF_1 = 0.1, MAF_2 = 0.3, MAF_3 = 0.3$				
$P(D)$ / MAF_4	0.1	0.2	0.3	0.4	0.5
0.3	0.840	0.000	0.000	0.000	0.000
0.4	0.065	0.000	0.000	0.000	0.000
0.5	0.000	0.000	0.000	0.000	0.000

	$h^2 = 0.15, MAF_1 = 0.1, MAF_2 = 0.3, MAF_3 = 0.4$				
$P(D)$ / MAF_4	0.1	0.2	0.3	0.4	0.5
0.4	0.005	0.000	0.000	0.000	0.000
0.5	0.000	0.000	0.000	0.000	0.000

	$h^2 = 0.15, MAF_1 = 0.1, MAF_2 = 0.3, MAF_3 = 0.5$				
$P(D)$ / MAF_4	0.1	0.2	0.3	0.4	0.5
0.5	0.001	0.000	0.000	0.000	0.000

	$h^2 = 0.15, MAF_1 = 0.1, MAF_2 = 0.4, MAF_3 = 0.4$				
$P(D)$ / MAF_4	0.1	0.2	0.3	0.4	0.5
0.4	0.150	0.000	0.000	0.000	0.000
0.5	0.020	0.000	0.000	0.000	0.000

	$h^2 = 0.15, MAF_1 = 0.1, MAF_2 = 0.4, MAF_3 = 0.5$				
$P(D)$ / MAF_4	0.1	0.2	0.3	0.4	0.5
0.5	0.010	0.000	0.000	0.000	0.000

	$h^2 = 0.15, MAF_1 = 0.1, MAF_2 = 0.5, MAF_3 = 0.5$				
$P(D)$ / MAF_4	0.1	0.2	0.3	0.4	0.5
0.5	0.000	0.000	0.000	0.000	0.000

	$h^2 = 0.15, MAF_1 = 0.2, MAF_2 = 0.2, MAF_3 = 0.2$				
$P(D)$ / MAF_4	0.1	0.2	0.3	0.4	0.5

0.2	1.000	0.000	0.000	0.000	0.000
0.3	1.000	0.000	0.000	0.000	0.000
0.4	0.155	0.000	0.000	0.000	0.000
0.5	0.000	0.000	0.000	0.000	0.000

	$h^2 = 0.15, MAF_1 = 0.2, MAF_2 = 0.2, MAF_3 = 0.3$				
$P(D)$ / MAF_4	0.1	0.2	0.3	0.4	0.5
0.3	1.000	0.085	0.000	0.000	0.000
0.4	0.000	0.180	0.000	0.000	0.000
0.5	0.000	0.040	0.000	0.000	0.000

	$h^2 = 0.15, MAF_1 = 0.1, MAF_2 = 0.2, MAF_3 = 0.4$				
$P(D)$ / MAF_4	0.1	0.2	0.3	0.4	0.5
0.4	0.055	0.005	0.000	0.000	0.000
0.5	0.050	0.010	0.000	0.000	0.000

	$h^2 = 0.15, MAF_1 = 0.2, MAF_2 = 0.5, MAF_3 = 0.5$				
$P(D)$ / MAF_4	0.1	0.2	0.3	0.4	0.5
0.5	0.015	0.000	0.000	0.000	0.000

	$h^2 = 0.15, MAF_1 = 0.2, MAF_2 = 0.3, MAF_3 = 0.3$				
$P(D)$ / MAF_4	0.1	0.2	0.3	0.4	0.5
0.3	1.000	0.080	0.000	0.000	0.000
0.4	0.875	0.000	0.000	0.000	0.000
0.5	0.870	0.025	0.000	0.000	0.000

	$h^2 = 0.15, MAF_1 = 0.2, MAF_2 = 0.3, MAF_3 = 0.4$				
$P(D)$ / MAF_4	0.1	0.2	0.3	0.4	0.5
0.4	0.985	0.000	0.000	0.000	0.000
0.5	0.430	0.000	0.000	0.000	0.000

	$h^2 = 0.15, MAF_1 = 0.2, MAF_2 = 0.3, MAF_3 = 0.5$				
$P(D)$ / MAF_4	0.1	0.2	0.3	0.4	0.5
0.5	0.001	0.000	0.000	0.000	0.000

	$h^2 = 0.15, MAF_1 = 0.2, MAF_2 = 0.4, MAF_3 = 0.4$				
$P(D)$ / MAF_4	0.1	0.2	0.3	0.4	0.5
0.4	0.250	0.000	0.000	0.000	0.000
0.5	0.000	0.000	0.000	0.000	0.000

	$h^2 = 0.15, MAF_1 = 0.2, MAF_2 = 0.4, MAF_3 = 0.5$				
$P(D)$ / MAF_4	0.1	0.2	0.3	0.4	0.5
0.5	0.000	0.000	0.000	0.000	0.000

	$h^2 = 0.15, MAF_1 = 0.2, MAF_2 = 0.5, MAF_3 = 0.5$				
$P(D)$ / MAF_4	0.1	0.2	0.3	0.4	0.5
0.5	0.000	0.000	0.000	0.000	0.000

	$h^2 = 0.15, MAF_1 = 0.3, MAF_2 = 0.3, MAF_3 = 0.3$				
$P(D)$ / MAF_4	0.1	0.2	0.3	0.4	0.5
0.3	0.995	0.000	0.000	0.000	0.000
0.4	0.770	0.015	0.000	0.000	0.000
0.5	0.985	0.000	0.000	0.000	0.000

	$h^2 = 0.15, MAF_1 = 0.3, MAF_2 = 0.3, MAF_3 = 0.4$				
$P(D)$ / MAF_4	0.1	0.2	0.3	0.4	0.5
0.4	0.980	0.000	0.000	0.000	0.000
0.5	0.975	0.000	0.000	0.000	0.000

	$h^2 = 0.15, MAF_1 = 0.3, MAF_2 = 0.3, MAF_3 = 0.5$				
$P(D)$ / MAF_4	0.1	0.2	0.3	0.4	0.5
0.5	0.060	0.000	0.000	0.000	0.000

	$h^2 = 0.15, MAF_1 = 0.3, MAF_2 = 0.4, MAF_3 = 0.4$				
$P(D)$ / MAF_4	0.1	0.2	0.3	0.4	0.5
0.4	0.060	0.000	0.000	0.000	0.000
0.5	0.080	0.000	0.000	0.000	0.000

	$h^2 = 0.15, MAF_1 = 0.3, MAF_2 = 0.4, MAF_3 = 0.5$				
$P(D)$ / MAF_4	0.1	0.2	0.3	0.4	0.5
0.5	0.000	0.000	0.000	0.000	0.000

	$h^2 = 0.15, MAF_1 = 0.3, MAF_2 = 0.5, MAF_3 = 0.5$				
$P(D)$ / MAF_4	0.1	0.2	0.3	0.4	0.5
0.5	0.000	0.000	0.000	0.000	0.000

	$h^2 = 0.15, MAF_1 = 0.4, MAF_2 = 0.4, MAF_3 = 0.4$				
$P(D)$ / MAF_4	0.1	0.2	0.3	0.4	0.5
0.4	0.800	0.000	0.000	0.000	0.000
0.5	0.060	0.000	0.000	0.000	0.000

	$h^2 = 0.15, MAF_1 = 0.4, MAF_2 = 0.4, MAF_3 = 0.5$				
$P(D)$ / MAF_4	0.1	0.2	0.3	0.4	0.5
0.5	0.000	0.000	0.000	0.000	0.000

	$h^2 = 0.15, MAF_1 = 0.4, MAF_2 = 0.5, MAF_3 = 0.5$				

MAF_4 \ $P(D)$	0.1	0.2	0.3	0.4	0.5
0.5	0.000	0.000	0.000	0.000	0.000
	$h^2 = 0.15, MAF_1 = 0.5, MAF_2 = 0.5 MAF_3 = 0.5$				
MAF_4 \ $P(D)$	0.1	0.2	0.3	0.4	0.5
0.5	0.000	0.000	0.000	0.000	0.000

Table S21: The detailed table of successful generation frequencies for 4-order eNME model with $h^2 = 0.2$.

	$h^2 = 0.2, MAF_1 = 0.1, MAF_2 = 0.1, MAF_3 = 0.1$				
MAF_4 \ $P(D)$	0.1	0.2	0.3	0.4	0.5
0.1	1.000	0.001	0.000	0.000	0.000
0.2	1.000	0.001	0.000	0.000	0.000
0.3	1.000	0.000	0.000	0.000	0.000
0.4	0.090	0.000	0.000	0.000	0.000
0.5	0.010	0.000	0.000	0.000	0.000

	$h^2 = 0.2, MAF_1 = 0.1, MAF_2 = 0.1, MAF_3 = 0.2$				
MAF_4 \ $P(D)$	0.1	0.2	0.3	0.4	0.5
0.2	1.000	0.002	0.000	0.000	0.000
0.3	1.000	0.000	0.000	0.000	0.000
0.4	0.250	0.000	0.000	0.000	0.000
0.5	0.015	0.000	0.000	0.000	0.000

	$h^2 = 0.2, MAF_1 = 0.1, MAF_2 = 0.1, MAF_3 = 0.3$				
MAF_4 \ $P(D)$	0.1	0.2	0.3	0.4	0.5
0.3	1.000	0.000	0.000	0.000	0.000
0.4	0.015	0.000	0.000	0.000	0.000
0.5	0.001	0.000	0.000	0.000	0.000

	$h^2 = 0.2, MAF_1 = 0.1, MAF_2 = 0.1, MAF_3 = 0.4$				
MAF_4 \ $P(D)$	0.1	0.2	0.3	0.4	0.5
0.4	0.005	0.000	0.000	0.000	0.000
0.5	0.000	0.000	0.000	0.000	0.000

	$h^2 = 0.2, MAF_1 = 0.1, MAF_2 = 0.1, MAF_3 = 0.5$				
MAF_4 \ $P(D)$	0.1	0.2	0.3	0.4	0.5
0.5	0.000	0.000	0.000	0.000	0.000

	$h^2 = 0.2, MAF_1 = 0.1, MAF_2 = 0.2, MAF_3 = 0.2$				
MAF_4 \ $P(D)$	0.1	0.2	0.3	0.4	0.5
0.2	1.000	0.990	0.000	0.000	0.000
0.3	1.000	0.515	0.000	0.000	0.000
0.4	1.000	0.000	0.000	0.000	0.000
0.5	0.930	0.000	0.000	0.000	0.000

	$h^2 = 0.2, MAF_1 = 0.1, MAF_2 = 0.2, MAF_3 = 0.3$				
MAF_4 \ $P(D)$	0.1	0.2	0.3	0.4	0.5
0.3	1.000	0.001	0.000	0.000	0.000
0.4	1.000	0.000	0.000	0.000	0.000
0.5	0.045	0.000	0.000	0.000	0.000

	$h^2 = 0.2, MAF_1 = 0.1, MAF_2 = 0.2, MAF_3 = 0.4$				
MAF_4 \ $P(D)$	0.1	0.2	0.3	0.4	0.5
0.4	0.210	0.000	0.000	0.000	0.000
0.5	0.005	0.000	0.000	0.000	0.000

	$h^2 = 0.2, MAF_1 = 0.1, MAF_2 = 0.2, MAF_3 = 0.5$				
MAF_4 \ $P(D)$	0.1	0.2	0.3	0.4	0.5
0.5	0.000	0.000	0.000	0.000	0.000

	$h^2 = 0.2, MAF_1 = 0.1, MAF_2 = 0.3, MAF_3 = 0.3$				
MAF_4 \ $P(D)$	0.1	0.2	0.3	0.4	0.5
0.3	0.780	0.000	0.000	0.000	0.000
0.4	0.010	0.000	0.000	0.000	0.000
0.5	0.000	0.000	0.000	0.000	0.000

	$h^2 = 0.2, MAF_1 = 0.1, MAF_2 = 0.3, MAF_3 = 0.4$				
MAF_4 \ $P(D)$	0.1	0.2	0.3	0.4	0.5
0.4	0.000	0.000	0.000	0.000	0.000
0.5	0.000	0.000	0.000	0.000	0.000

	$h^2 = 0.2, MAF_1 = 0.1, MAF_2 = 0.3, MAF_3 = 0.5$				
MAF_4 \ $P(D)$	0.1	0.2	0.3	0.4	0.5
0.5	0.000	0.000	0.000	0.000	0.000

	$h^2 = 0.2, MAF_1 = 0.1, MAF_2 = 0.4, MAF_3 = 0.4$				
MAF_4 \ $P(D)$	0.1	0.2	0.3	0.4	0.5
0.4	0.205	0.000	0.000	0.000	0.000
0.5	0.015	0.000	0.000	0.000	0.000

	$h^2 = 0.2, MAF_1 = 0.1, MAF_2 = 0.4, MAF_3 = 0.5$				
MAF_4 \ $P(D)$	0.1	0.2	0.3	0.4	0.5
0.5	0.000	0.000	0.000	0.000	0.000

	$h^2 = 0.2, MAF_1 = 0.1, MAF_2 = 0.5, MAF_3 = 0.5$				
MAF_4 \ $P(D)$	0.1	0.2	0.3	0.4	0.5
0.5	0.005	0.000	0.000	0.000	0.000

	$h^2 = 0.2, MAF_1 = 0.2, MAF_2 = 0.2, MAF_3 = 0.2$				
MAF_4 \ $P(D)$	0.1	0.2	0.3	0.4	0.5

0.2	1.000	0.000	0.000	0.000	0.000
0.3	1.000	0.000	0.000	0.000	0.000
0.4	0.150	0.000	0.000	0.000	0.000
0.5	0.000	0.000	0.000	0.000	0.000

	$h^2 = 0.2, MAF_1 = 0.2, MAF_2 = 0.2, MAF_3 = 0.3$				
MAF_4 \ $P(D)$	0.1	0.2	0.3	0.4	0.5
0.3	1.000	0.000	0.000	0.000	0.000
0.4	0.000	0.000	0.000	0.000	0.000
0.5	0.000	0.000	0.000	0.000	0.000

	$h^2 = 0.2, MAF_1 = 0.1, MAF_2 = 0.2, MAF_3 = 0.4$				
MAF_4 \ $P(D)$	0.1	0.2	0.3	0.4	0.5
0.4	0.010	0.015	0.000	0.000	0.000
0.5	0.010	0.020	0.000	0.000	0.000

	$h^2 = 0.2, MAF_1 = 0.2, MAF_2 = 0.5, MAF_3 = 0.5$				
MAF_4 \ $P(D)$	0.1	0.2	0.3	0.4	0.5
0.5	0.020	0.000	0.000	0.000	0.000

	$h^2 = 0.2, MAF_1 = 0.2, MAF_2 = 0.3, MAF_3 = 0.3$				
MAF_4 \ $P(D)$	0.1	0.2	0.3	0.4	0.5
0.3	1.000	0.130	0.000	0.000	0.000
0.4	0.945	0.030	0.000	0.000	0.000
0.5	0.975	0.005	0.000	0.000	0.000

	$h^2 = 0.2, MAF_1 = 0.2, MAF_2 = 0.3, MAF_3 = 0.4$				
MAF_4 \ $P(D)$	0.1	0.2	0.3	0.4	0.5
0.4	1.000	0.130	0.000	0.000	0.000
0.5	0.310	0.000	0.000	0.000	0.000

	$h^2 = 0.2, MAF_1 = 0.2, MAF_2 = 0.3, MAF_3 = 0.5$				
MAF_4 \ $P(D)$	0.1	0.2	0.3	0.4	0.5
0.5	0.015	0.000	0.000	0.000	0.000

	$h^2 = 0.2, MAF_1 = 0.2, MAF_2 = 0.4, MAF_3 = 0.4$				
MAF_4 \ $P(D)$	0.1	0.2	0.3	0.4	0.5
0.4	0.465	0.000	0.000	0.000	0.000
0.5	0.020	0.000	0.000	0.000	0.000

	$h^2 = 0.2, MAF_1 = 0.2, MAF_2 = 0.4, MAF_3 = 0.5$				
MAF_4 \ $P(D)$	0.1	0.2	0.3	0.4	0.5
0.5	0.000	0.000	0.000	0.000	0.000

	$h^2 = 0.2, MAF_1 = 0.2, MAF_2 = 0.5, MAF_3 = 0.5$				
MAF_4 \ $P(D)$	0.1	0.2	0.3	0.4	0.5
0.5	0.000	0.000	0.000	0.000	0.000

	$h^2 = 0.2, MAF_1 = 0.3, MAF_2 = 0.3, MAF_3 = 0.3$				
MAF_4 \ $P(D)$	0.1	0.2	0.3	0.4	0.5
0.3	0.995	0.010	0.000	0.000	0.000
0.4	0.300	0.030	0.000	0.000	0.000
0.5	0.685	0.000	0.000	0.000	0.000

	$h^2 = 0.2, MAF_1 = 0.3, MAF_2 = 0.3, MAF_3 = 0.4$				
MAF_4 \ $P(D)$	0.1	0.2	0.3	0.4	0.5
0.4	1.000	0.000	0.000	0.000	0.000
0.5	1.000	0.000	0.000	0.000	0.000

	$h^2 = 0.2, MAF_1 = 0.3, MAF_2 = 0.3, MAF_3 = 0.5$				
MAF_4 \ $P(D)$	0.1	0.2	0.3	0.4	0.5
0.5	0.015	0.000	0.000	0.000	0.000

	$h^2 = 0.2, MAF_1 = 0.3, MAF_2 = 0.4, MAF_3 = 0.4$				
MAF_4 \ $P(D)$	0.1	0.2	0.3	0.4	0.5
0.4	0.765	0.000	0.000	0.000	0.000
0.5	0.185	0.000	0.000	0.000	0.000

	$h^2 = 0.2, MAF_1 = 0.3, MAF_2 = 0.4, MAF_3 = 0.5$				
MAF_4 \ $P(D)$	0.1	0.2	0.3	0.4	0.5
0.5	0.000	0.000	0.000	0.000	0.000

	$h^2 = 0.2, MAF_1 = 0.3, MAF_2 = 0.5, MAF_3 = 0.5$				
MAF_4 \ $P(D)$	0.1	0.2	0.3	0.4	0.5
0.5	0.000	0.000	0.000	0.000	0.000

	$h^2 = 0.2, MAF_1 = 0.4, MAF_2 = 0.4, MAF_3 = 0.4$				
MAF_4 \ $P(D)$	0.1	0.2	0.3	0.4	0.5
0.4	0.720	0.000	0.000	0.000	0.000
0.5	0.435	0.000	0.000	0.000	0.000

	$h^2 = 0.2, MAF_1 = 0.4, MAF_2 = 0.4, MAF_3 = 0.5$				
MAF_4 \ $P(D)$	0.1	0.2	0.3	0.4	0.5
0.5	0.005	0.000	0.000	0.000	0.000

	$h^2 = 0.2, MAF_1 = 0.4, MAF_2 = 0.5, MAF_3 = 0.5$				

MAF_4 \ $P(D)$	0.1	0.2	0.3	0.4	0.5
0.5	0.005	0.000	0.000	0.000	0.000
	$h^2 = 0.2, MAF_1 = 0.5, MAF_2 = 0.5 MAF_3 = 0.5$				
MAF_4 \ $P(D)$	0.1	0.2	0.3	0.4	0.5
0.5	0.005	0.000	0.000	0.000	0.000

Table S22: The detailed table of successful generation frequencies for 4-order eNME model with $h^2 = 0.3$.

$h^2 = 0.3, MAF_1 = 0.1, MAF_2 = 0.1, MAF_3 = 0.1$

$P(D)$ / MAF_4	0.1	0.2	0.3	0.4	0.5
0.1	1.000	0.001	0.000	0.000	0.000
0.2	1.000	0.000	0.000	0.000	0.000
0.3	1.000	0.000	0.000	0.000	0.000
0.4	0.000	0.000	0.000	0.000	0.000
0.5	0.000	0.000	0.000	0.000	0.000

$h^2 = 0.3, MAF_1 = 0.1, MAF_2 = 0.1, MAF_3 = 0.2$

$P(D)$ / MAF_4	0.1	0.2	0.3	0.4	0.5
0.2	1.000	0.002	0.000	0.000	0.000
0.3	1.000	0.000	0.000	0.000	0.000
0.4	0.250	0.000	0.000	0.000	0.000
0.5	0.015	0.000	0.000	0.000	0.000

$h^2 = 0.3, MAF_1 = 0.1, MAF_2 = 0.1, MAF_3 = 0.3$

$P(D)$ / MAF_4	0.1	0.2	0.3	0.4	0.5
0.3	1.000	0.000	0.000	0.000	0.000
0.4	0.015	0.000	0.000	0.000	0.000
0.5	0.000	0.000	0.000	0.000	0.000

$h^2 = 0.3, MAF_1 = 0.1, MAF_2 = 0.1, MAF_3 = 0.4$

$P(D)$ / MAF_4	0.1	0.2	0.3	0.4	0.5
0.4	0.000	0.000	0.000	0.000	0.000
0.5	0.000	0.000	0.000	0.000	0.000

$h^2 = 0.3, MAF_1 = 0.1, MAF_2 = 0.1, MAF_3 = 0.5$

$P(D)$ / MAF_4	0.1	0.2	0.3	0.4	0.5
0.5	0.000	0.000	0.000	0.000	0.000

$h^2 = 0.3, MAF_1 = 0.1, MAF_2 = 0.2, MAF_3 = 0.2$

$P(D)$ / MAF_4	0.1	0.2	0.3	0.4	0.5
0.2	1.000	0.975	0.000	0.000	0.000
0.3	1.000	0.405	0.000	0.000	0.000
0.4	0.995	0.000	0.000	0.000	0.000
0.5	0.890	0.000	0.000	0.000	0.000

$h^2 = 0.3, MAF_1 = 0.1, MAF_2 = 0.2, MAF_3 = 0.3$

$P(D)$ / MAF_4	0.1	0.2	0.3	0.4	0.5
0.3	1.000	0.001	0.000	0.000	0.000
0.4	1.000	0.000	0.000	0.000	0.000
0.5	0.060	0.000	0.000	0.000	0.000

$h^2 = 0.3, MAF_1 = 0.1, MAF_2 = 0.2, MAF_3 = 0.4$

$P(D)$ / MAF_4	0.1	0.2	0.3	0.4	0.5
0.4	0.080	0.000	0.000	0.000	0.000
0.5	0.000	0.000	0.000	0.000	0.000

$h^2 = 0.3, MAF_1 = 0.1, MAF_2 = 0.2, MAF_3 = 0.5$

$P(D)$ / MAF_4	0.1	0.2	0.3	0.4	0.5
0.5	0.000	0.000	0.000	0.000	0.000

$h^2 = 0.3, MAF_1 = 0.1, MAF_2 = 0.3, MAF_3 = 0.3$

$P(D)$ / MAF_4	0.1	0.2	0.3	0.4	0.5
0.3	0.640	0.000	0.000	0.000	0.000
0.4	0.000	0.000	0.000	0.000	0.000
0.5	0.000	0.000	0.000	0.000	0.000

$h^2 = 0.3, MAF_1 = 0.1, MAF_2 = 0.3, MAF_3 = 0.4$

$P(D)$ / MAF_4	0.1	0.2	0.3	0.4	0.5
0.4	0.000	0.000	0.000	0.000	0.000
0.5	0.000	0.000	0.000	0.000	0.000

$h^2 = 0.3, MAF_1 = 0.1, MAF_2 = 0.3, MAF_3 = 0.5$

$P(D)$ / MAF_4	0.1	0.2	0.3	0.4	0.5
0.5	0.000	0.000	0.000	0.000	0.000

$h^2 = 0.3, MAF_1 = 0.1, MAF_2 = 0.4, MAF_3 = 0.4$

$P(D)$ / MAF_4	0.1	0.2	0.3	0.4	0.5
0.4	0.205	0.000	0.000	0.000	0.000
0.5	0.000	0.000	0.000	0.000	0.000

$h^2 = 0.3, MAF_1 = 0.1, MAF_2 = 0.4, MAF_3 = 0.5$

$P(D)$ / MAF_4	0.1	0.2	0.3	0.4	0.5
0.5	0.000	0.000	0.000	0.000	0.000

$h^2 = 0.3, MAF_1 = 0.1, MAF_2 = 0.5, MAF_3 = 0.5$

$P(D)$ / MAF_4	0.1	0.2	0.3	0.4	0.5
0.5	0.000	0.000	0.000	0.000	0.000

$h^2 = 0.3, MAF_1 = 0.2, MAF_2 = 0.2, MAF_3 = 0.2$

$P(D)$ / MAF_4	0.1	0.2	0.3	0.4	0.5

0.2	1.000	0.000	0.000	0.000	0.000
0.3	1.000	0.030	0.000	0.000	0.000
0.4	0.105	0.000	0.000	0.000	0.000
0.5	0.000	0.000	0.000	0.000	0.000

	$h^2 = 0.3, MAF_1 = 0.2, MAF_2 = 0.2, MAF_3 = 0.3$				
$P(D)$ / MAF_4	0.1	0.2	0.3	0.4	0.5
0.3	1.000	0.040	0.000	0.000	0.000
0.4	0.015	0.000	0.000	0.000	0.000
0.5	0.000	0.000	0.000	0.000	0.000

	$h^2 = 0.3, MAF_1 = 0.1, MAF_2 = 0.2, MAF_3 = 0.4$				
$P(D)$ / MAF_4	0.1	0.2	0.3	0.4	0.5
0.4	0.000	0.000	0.000	0.000	0.000
0.5	0.000	0.000	0.000	0.000	0.000

	$h^2 = 0.3, MAF_1 = 0.2, MAF_2 = 0.5, MAF_3 = 0.5$				
$P(D)$ / MAF_4	0.1	0.2	0.3	0.4	0.5
0.5	0.000	0.000	0.000	0.000	0.000

	$h^2 = 0.3, MAF_1 = 0.2, MAF_2 = 0.3, MAF_3 = 0.3$				
$P(D)$ / MAF_4	0.1	0.2	0.3	0.4	0.5
0.3	1.000	0.175	0.000	0.000	0.000
0.4	0.720	0.125	0.000	0.000	0.000
0.5	0.035	0.010	0.000	0.000	0.000

	$h^2 = 0.3, MAF_1 = 0.2, MAF_2 = 0.3, MAF_3 = 0.4$				
$P(D)$ / MAF_4	0.1	0.2	0.3	0.4	0.5
0.4	0.980	0.000	0.000	0.000	0.000
0.5	0.215	0.000	0.000	0.000	0.000

	$h^2 = 0.3, MAF_1 = 0.2, MAF_2 = 0.3, MAF_3 = 0.5$				
$P(D)$ / MAF_4	0.1	0.2	0.3	0.4	0.5
0.5	0.020	0.000	0.000	0.000	0.000

	$h^2 = 0.3, MAF_1 = 0.2, MAF_2 = 0.4, MAF_3 = 0.4$				
$P(D)$ / MAF_4	0.1	0.2	0.3	0.4	0.5
0.4	0.800	0.000	0.000	0.000	0.000
0.5	0.555	0.000	0.000	0.000	0.000

	$h^2 = 0.3, MAF_1 = 0.2, MAF_2 = 0.4, MAF_3 = 0.5$				
$P(D)$ / MAF_4	0.1	0.2	0.3	0.4	0.5
0.5	0.000	0.000	0.000	0.000	0.000

	$h^2 = 0.3, MAF_1 = 0.2, MAF_2 = 0.5, MAF_3 = 0.5$				
$P(D)$ / MAF_4	0.1	0.2	0.3	0.4	0.5
0.5	0.000	0.000	0.000	0.000	0.000

	$h^2 = 0.3, MAF_1 = 0.3, MAF_2 = 0.3, MAF_3 = 0.3$				
$P(D)$ / MAF_4	0.1	0.2	0.3	0.4	0.5
0.3	0.930	0.150	0.000	0.000	0.000
0.4	0.010	0.310	0.000	0.000	0.000
0.5	0.060	0.075	0.000	0.000	0.000

	$h^2 = 0.3, MAF_1 = 0.3, MAF_2 = 0.3, MAF_3 = 0.4$				
$P(D)$ / MAF_4	0.1	0.2	0.3	0.4	0.5
0.4	1.000	0.000	0.000	0.000	0.000
0.5	0.920	0.000	0.000	0.000	0.000

	$h^2 = 0.3, MAF_1 = 0.3, MAF_2 = 0.3, MAF_3 = 0.5$				
$P(D)$ / MAF_4	0.1	0.2	0.3	0.4	0.5
0.5	0.010	0.000	0.000	0.000	0.000

	$h^2 = 0.3, MAF_1 = 0.3, MAF_2 = 0.4, MAF_3 = 0.4$				
$P(D)$ / MAF_4	0.1	0.2	0.3	0.4	0.5
0.4	0.740	0.000	0.000	0.000	0.000
0.5	0.160	0.000	0.000	0.000	0.000

	$h^2 = 0.3, MAF_1 = 0.3, MAF_2 = 0.4, MAF_3 = 0.5$				
$P(D)$ / MAF_4	0.1	0.2	0.3	0.4	0.5
0.5	0.000	0.000	0.000	0.000	0.000

	$h^2 = 0.3, MAF_1 = 0.3, MAF_2 = 0.5, MAF_3 = 0.5$				
$P(D)$ / MAF_4	0.1	0.2	0.3	0.4	0.5
0.5	0.000	0.000	0.000	0.000	0.000

	$h^2 = 0.3, MAF_1 = 0.4, MAF_2 = 0.4, MAF_3 = 0.4$				
$P(D)$ / MAF_4	0.1	0.2	0.3	0.4	0.5
0.4	0.720	0.000	0.000	0.000	0.000
0.5	0.435	0.000	0.000	0.000	0.000

	$h^2 = 0.3, MAF_1 = 0.4, MAF_2 = 0.4, MAF_3 = 0.5$				
$P(D)$ / MAF_4	0.1	0.2	0.3	0.4	0.5
0.5	0.000	0.000	0.000	0.000	0.000

	$h^2 = 0.3, MAF_1 = 0.4, MAF_2 = 0.5, MAF_3 = 0.5$				

$P(D)$ / MAF_4	0.1	0.2	0.3	0.4	0.5
0.5	0.000	0.000	0.000	0.000	0.000
	$h^2 = 0.3, MAF_1 = 0.5, MAF_2 = 0.5 MAF_3 = 0.5$				
$P(D)$ / MAF_4	0.1	0.2	0.3	0.4	0.5
0.5	0.000	0.000	0.000	0.000	0.000
